# Reducing phenolic off-flavors through CRISPR-based gene editing of the *FDC1* gene in *Saccharomyces cerevisiae* x *Saccharomyces eubayanus* hybrid lager beer yeasts

**DOI:** 10.1371/journal.pone.0209124

**Published:** 2019-01-09

**Authors:** Stijn Mertens, Brigida Gallone, Jan Steensels, Beatriz Herrera-Malaver, Jeroen Cortebeek, Robbe Nolmans, Veerle Saels, Valmik K. Vyas, Kevin J. Verstrepen

**Affiliations:** 1 Laboratory for Genetics and Genomics, Centre of Microbial and Plant Genetics (CMPG), KU Leuven, Leuven, Belgium; 2 Laboratory for Systems Biology, VIB Centre for Microbiology, Bio-Incubator, Leuven, Belgium; 3 Leuven Institute for Beer Research, KU Leuven, Bio-Incubator, Leuven, Belgium; 4 Department of Plant Systems Biology, VIB, Gent, Belgium; 5 Department of Plant Biotechnology and Bioinformatics, Ghent University, Gent, Belgium; 6 Whitehead Institute for Biomedical Research, Cambridge, Massachusetts, United States of America; University of Strasbourg, FRANCE

## Abstract

Today’s beer market is challenged by a decreasing consumption of traditional beer styles and an increasing consumption of specialty beers. In particular, lager-type beers (pilsner), characterized by their refreshing and unique aroma and taste, yet very uniform, struggle with their sales. The development of novel variants of the common lager yeast, the interspecific hybrid *Saccharomyces pastorianus*, has been proposed as a possible solution to address the need of product diversification in lager beers. Previous efforts to generate new lager yeasts through hybridization of the ancestral parental species (*S*. *cerevisiae* and *S*. *eubayanus*) yielded strains with an aromatic profile distinct from the natural biodiversity. Unfortunately, next to the desired properties, these novel yeasts also inherited unwanted characteristics. Most notably is their phenolic off-flavor (POF) production, which hampers their direct application in the industrial production processes. Here, we describe a CRISPR-based gene editing strategy that allows the systematic and meticulous introduction of a natural occurring mutation in the *FDC1* gene of genetically complex industrial *S*. *cerevisiae* strains, *S*. *eubayanus* yeasts and interspecific hybrids. The resulting cisgenic POF^-^ variants show great potential for industrial application and diversifying the current lager beer portfolio.

## Introduction

Although interspecific hybridization (i.e. hybridization between two different species) is believed to be rare in nature, next generation sequencing recently revealed the presence of several interspecific hybrid yeasts within the *Saccharomyces* yeast clade [1–3]. While some hybrids have occasionally been isolated from natural habitats, most isolates were obtained from man-made industrial environments [4, 5]. The best-known and most studied example of such interspecific hybrid is *Saccharomyces pastorianus*, the yeast species used for lager beer production. This hybrid yeast originated from a cross between the commonly used ale beer yeast *Saccharomyces cerevisiae* and the cold-tolerant species *Saccharomyces eubayanus* [6–8]. The resulting *S*. *pastorianus* hybrid combines the ability of *S*. *cerevisiae* to efficiently ferment sugars in beer wort with the cold-tolerance of *S*. *eubayanus*, making it the ideal yeast to perform lager beer fermentations which are typically performed at lower temperatures [6, 9, 10]. Besides *S*. *pastorianus*, other hybrid types are associated with industrial environments, such as *S*. *cerevisiae* and *S*. *kudriavzevii* hybrids (isolated from ale beer and wine fermentations), and *S*. *cerevisiae* X *S*. *uvarum* hybrids, which are sometimes isolated from wine and beer fermentations [1, 11–13].

The discovery of an increasing number of interspecific hybrids in industrial fermentation processes inspired researchers to mimic the hybridization in the lab, often with the aim of generating new variants that would expand the existing spectrum of industrial yeasts [8]. Hybridization between different strains of *S*. *cerevisiae* strains has proven an effective method to generate new variants with interesting industrial properties [14–16], and the ability to include non-*cerevisiae* strains in the breeding schemes further broadens the gene pool and thus the phenotypic diversity of the resulting hybrids. Over the past years, multiple reports describe hybridization between *S*. *cerevisiae* strains and *S*. *uvarum* [17–19], *S*. *eubayanus* [19–24], *S*. *kudriavzevii* [25, 26] or *S*. *arboricola* [19]. The newly generated interspecific hybrids often show hybrid vigor in their fermentative capacity, broadened temperature tolerance and/or diversified metabolite and aroma profiles. Interspecific hybrids therefore open new routes to address changes in the global fermented beverage market, including the increasing demand for low alcohol, high-flavor and unique products [8, 27].

While newly formed interspecific hybrids combine interesting characteristics of their respective parental species, they can also inherit undesired phenotypes that impede their direct implementation in industrial production processes. More specifically, the non-*cerevisiae* strains are less adapted to industrial fermentations and as a result they often perform poorly in the specific stress conditions imposed by industrial environments. Moreover, the wild yeasts often produce certain undesirable aroma compounds. Arguably the most important drawback of the wild species is the production of phenolic off-flavors (POF), most notably 4-vinyl guaiacol (4VG) [19, 21, 23]. In beer, 4VG is usually an undesirable yeast metabolite that imposes a very distinct spicy, clove-like flavor. It is produced by yeast through the bioconversion of ferulic acid, present in the endosperm of the malt and barley, to its decarboxylated derivative, 4VG [28, 29]. The genetic underpinnings of this phenotype are well-described, and involve the action of Fdc1p and Pad1p [28, 29]. The first decarboxylates ferulic acid, while the latter provides a prenylated flavin-mononucleotide (FMN) cofactor of Fdc1p, required for its function.

Several successful strategies have been described to obtain artificial interspecific yeast hybrids that are POF^-^. First, after the hybrid has been formed, an additional step consisting of a backcross to the POF^-^ *S*. *cerevisiae* parent effectively removes the phenotype. However, newly formed interspecific hybrids are usually sterile due to the postzygotic barriers between members of the *Saccharomyces* clade, which limit interspecific hybrids to a vegetative lifestyle [14, 30]. Interestingly though, recent publications have found a way to circumvent this hybrid sterility. A first approach is the generation of allotetraploid interspecific hybrids via rare mating of a diploid *S*. *cerevisiae* strain with a diploid non-*cerevisiae* yeast cell [18, 20, 31]. The resulting allotetraploid interspecific hybrids can form viable allodiploid spores which could be used for backcrossing with spores of its POF^-^ parental strain or with another yeast. This approach allowed Krogerus and coworkers to generate a POF^-^ interspecific yeast hybrid, combining genetic material of three parental strains [20]. Nevertheless, this approach also has some major drawbacks. It relies on a rather complex breeding scheme, on the generation of auxotrophic mutants of the candidate parental yeasts and on two consecutive rounds of breeding that require extensive screening of the segregants.

A second approach involves direct modification of the non-*cerevisiae* parent to eliminate the POF phenotype using mutagenesis [32]. Segregants of the *S*. *eubayanus* parental strain are subjected to UV mutagenesis and subsequently screened to identify POF^-^ mutants that can be directly applied in a breeding scheme. However, this strategy also has substantial limitations, most notably the large screenings required to identify positive mutants and the risk of off-target mutations with potential undesired phenotypic effects. Despite the availability of a high-throughput screening method for POF production [33], identification of a POF^-^ mutant with no or a very limited number of mutations in other genes would require a screening setup with an even higher throughput.

The Clustered Regularly Interspaced Short Palindromic Repeats (CRISPR) and CRISPR associated protein (Cas9)-based genome editing technology offers a new tool to modify phenotypes of industrial *Saccharomyces* yeasts [34, 35]. For example, a CRISPR-based genome editing strategy successfully reduced urea production in wine yeasts [36] and introduced the hops monoterpenes biosynthesis pathway in an ale brewing yeast, yielding yeast cells that produce certain hop aromas [37]. Moreover, a recent publication describes an optimized CRISPR strategy to alter the genome of the industrial *S*. *pastorianus* yeasts CBS1483 and W34/70, which allows to efficiently alter specific phenotypes such as ester production, by knocking out the responsible genes [38].

While CRISPR-based technologies demonstrate a broad spectrum of potential applications, the legislation surrounding the use of cisgenic gene-edited organisms differs widely between different countries across the planet. A recent EU ruling states that these organisms should follow the same guidelines as other genetically-modified organisms. By contrast, other countries, including Brazil, USA, Japan and Argentina, have installed specific guidelines for the use of CRISPR-based gene editing that in some cases allow such modified organisms to be used without following the GM legislation, which greatly increases their industrial potential [39–41].

In this study, we report a CRISPR-based gene editing strategy to develop cisgenic POF^-^variants of genetically complex industrial yeasts and interspecific hybrids by introducing a naturally occurring loss-of-function mutation in the *FDC1* gene. We applied and optimized this strategy for *S*. *cerevisiae* strains with varying ploidy, a non-*cerevisiae* species (*S*. *eubayanus*), and newly developed interspecific lager yeasts. We show that our strategy allows the introduction of homozygous mutations, resulting in cisgenic mutants that lost the ability to produce POF without undesirable side effects.

## Material and methods

### Yeast strains used in this study

Yeast parental strains for the generation of interspecific hybrids were selected from a collection of 301 industrial and wild *Saccharomyces* yeasts, previously characterized by Steensels and coworkers [16]. *S*. *cerevisiae* strains SA003 and BE011 were selected based on their POF pheno- and genotype, as well as for their ability to form viable spores [16, 42]. Additionally, *S*. *eubayanus* strains WL2022 (NPCC1286 [43]) and WL024 (NPCC1292 [43]) were selected based on their temperature tolerance and sporulation efficiency and viability. *S*. *cerevisiae* strains BE002, BE014, BE020 and BE074 were selected as candidate strains to test the proposed CRISPR-based genome editing strategy in industrially relevant *S*. *cerevisiae* strains. An overview of the used yeast strains is given in Table 1.

**Table 1:** Overview of the yeast strains used in this study.

Strain	Species	Industry	Origin
S288C (n)	*S*. *cerevisiae*	Lab	[44]
S288C(n)_A	*Gene edited S*. *cerevisiae*	Lab	This study
S288C (2n)	*S*. *cerevisiae*	Lab	[44]
S288C(2n)_A	*Gene edited S*. *cerevisiae*	Lab	This study
SP003	*S*. *cerevisiae*	Saké	Japan
BE011	*S*. *cerevisiae*	Beer	Belgium
BE002	*S*. *cerevisiae*	Beer	Bulgaria
BE002_A	Gene edited *S*. *cerevisiae*	Lab	This study
BE014	*S*. *cerevisiae*	Beer	Belgium
BE014_A	Gene edited *S*. *cerevisiae*	Lab	This study
BE014_B	Gene edited *S*. *cerevisiae*	Lab	This study
BE014_C	Gene edited *S*. *cerevisiae*	Lab	This study
BE020	*S*. *cerevisiae*	Beer	Belgium
BE020_A	Gene edited *S*. *cerevisiae*	Lab	This study
BE020_B	Gene edited *S*. *cerevisiae*	Lab	This study
BE020_C	Gene edited *S*. *cerevisiae*	Lab	This study
BE074	*S*. *cerevisiae*	Beer	Germany
BE074_A	Gene edited *S*. *cerevisiae*	Lab	This study
WL022 (NPCC1286) ^a^	*S*. *eubayanus*	Wild	Argentina [43]
WL022_A	Gene edited *S*. *eubayanus*	Lab	This study
WL024 (NPCC1292) ^a^	*S*. *eubayanus*	Wild	Argentina [43]
WL024_A	Gene edited *S*. *eubayanus*	Lab	This study
W34/70	*S*. *pastorianus*	Lager	Germany
NCYC2888 ^b^	*S*. *mikatae*	Wild	Japan
H1	Interspecific hybrid (BE011 X WL022)	Lab	This study
H1_A	Gene edited H1	Lab	This study
H1_B	Gene edited H1	Lab	This study
H1_C	Gene edited H1	Lab	This study
H1_D	Gene edited H1	Lab	This study
H2	Interspecific hybrid (SP003 X WL022)	Lab	This study
H2_A	Gene edited H2	Lab	This study
H2_B	Gene edited H2	Lab	This study
H2_C	Gene edited H2	Lab	This study
H2_D	Gene edited H2	Lab	This study

^a^NPCC: North Patagonian Culture Collection, Neuquén, Argentina

^b^NCYC: National Collection of Yeast Cultures, Quadram Institute Bioscience, Norwich, UK.

### Interspecific hybrid generation through spore to spore mating and hybrid-state confirmation

Interspecific yeast hybrids were generated by the previously described spore to spore mating technique [23]. First, parental strains were subjected to a random spore isolation protocol [15]. Next, a micromanipulator (MSM-singer instruments) was used to pair two spores on a YPD2% agar plate (2%[wt vol^-1^] Bacto peptone,1%[wt vol^-1^] yeast extract, 1.5%[wt vol^-1^] and 2%[wt vol^-1^] glucose), one from each parental strain. After six to eight hours of incubation at room temperature, the formation of a zygote (‘Shmoo’) was investigated. Possible hybrids were further purified by restreaking (3x) the strains on synthetic 12°P wort medium (Light spray malt extract: 13%[wt vol^-1^]; Agar: 1.5%[wt vol^-1^]). The hybrid nature of the possible hybrids was confirmed via a species specific multiplex PCR, as described in previous research [23] (S4 Fig). PCR-confirmed interspecific hybrids were prolonged stored at -80°C to ensure strain purity. Afterwards, generated hybrids were genetically stabilized according to the previously reported stabilization protocol [23].

### CRISPR-Cas9 based gene editing

#### Description plasmid

A *S*. *cerevisiae* compatible version of the *Candida albicans* solo vector CRISPR system, previously described by Vyas and coworkers [45], was used as a platform for the CRISPR-based gene editing [46]. The *S*. *cerevisiae* and *C*. *albicans* codon optimized Cas9 endonuclease (CaCas9, where the use of the ‘CUG’ leucine codon is avoided, which is predominantly translated as serine by CTG clade species like *C*. *albicans* and *S*. *cerevisiae* [45]), as well as the single guide (sg)RNA were introduced into the yeast shuttle vector pR5416, which provides a CEN/ARS sequence for plasmid maintenance in yeast [47]. Both the CaCas9 and the sgRNA are preceded by a constitutive promoter (respectively *TEF1p* and *SNR52p*). Species specific sgRNA sequences (*S*. *cerevisiae* 5-GGCAAGTACTTACAAACGTA-3’; *S*. *eubayanus* 5’-GGCAAGTATTTGCAAACGTA-3’) were cloned into the vector as described previously [45].

#### Repair templates

Double stranded (ds) DNA oligos were created as repair template for the homology directed repair of the induced DSB. Each of the repair templates are 100 nucleotides long, and are centered around the induced DSB in the yeast DNA and contained desired mutations in its sequence. The 100 nucleotides long repair templates were generated via a PCR fill-in of two 60bp long primers with an overlap of 20 nucleotides at their three prime end (primer sequence, see S10 Table).

#### Transformation

A standard lithium acetate-based yeast transformation protocol was used to transform both the CRISPR plasmid, as well as the repair template into the target strains [33]. Firstly, yeast was grown for one overnight in 5 mL YPD2% growth medium at 30°C, 200 rpm, after which 1 mL of the pregrowth was transferred to 50 mL YPD2% growth medium and incubated for an extra 4hours (30°C, 200 rpm). Next, the yeast cell culture was centrifuged (3 minutes at 3000 rpm) and cells were resuspended into 200 μL 0.1 M lithium acetate solution. After 10 minutes incubation at room temperature, 50 μL of the cell culture was mixed with 500 ng plasmid, in which the corresponding sgRNA was cloned and 5 to 25 μg (adjusted protocol) repair template DNA, 300 μL PLI (142 M Polyethylene glycol, 0.12 M lithium acetate, 0.01 M Tris (pH7.5) and 0.001M EDTA) and 5 μL salmon sperm DNA (1mg.mL^-1^) and incubated for 30 minutes at 42°C. Cells were centrifuged (3 minutes at 3000 rpm) and resuspended in fresh YPD2%, after which cells were recuperated for one overnight on YPD2% agar plates at 30°C. Selection of plasmid-containing cells was done via replica plating onto selective agar plates (YPD2% + clonat (0.2 μg.mL^-1^), followed by a 2 day incubation at 30°C. Growing colonies were subjected to a second round of selection on YPD2% + clonat agar plates or immediately plated on YPD2% agar plates (adjusted protocol). After selection, strains were grown for three consecutive rounds onto YPD2% agar plates in order to induce plasmid loss, prior to long term storage at -80°C.

Introduction of the correct mutation was determined via Sanger sequencing with species-specific primers (see S10 Table for an overview of used primers).

### Absorbance-based POF measurement

The ability of the yeasts to produce POF was tested via the absorbance based detection method, described previously [33]. Yeasts were inoculated in 150 μL liquid YPD2% growth medium, supplemented with 100 mg.L^-1^ ferulic acid in a 96 well plate. In each plate, a POF^-^ (W34/70) and a POF^+^ (*S*. *mikatae* NCYC2888) control were included. 96-well plates were sealed with an aluminum sticker and incubated for 5 days at 30°C, 200 rpm. After centrifugation (3 min, 3000 rpm), 100 μL of the supernatant was transferred to a new 96 well plate and remaining concentration of ferulic acid was measured at a wavelength of 325 nm (Tecan Infinite 200 PRO, Switzerland). Yeasts were regarded as POF^-^ if the absorbance at 325 nm was above the lower limit of the 90% confidence interval of the POF^-^ control (W34/70).

### Laboratory-scale lager fermentations

Lab scale fermentations were performed according a previously described protocol [23]. First, yeast was propagated by inoculation into 5 mL YPD2% medium at room temperature and 300 rpm. After 16 h of incubation, 1 mL of the culture was transferred to 50 mL 4% yeast extract-peptone-maltose (YPM;2%[wt vol^-1^] Bacto peptone,1%[wt vol^-1^] yeast extract, and 4%[wt vol^-1^] maltose) in a 250 mL Erlenmeyer flask and incubated at 20°C and 200 rpm for 3 overnights.

Cell concentration of the pregrowths was measured (BioRad, TC20 automated cell counter, USA), and the calculated amount of cells was used to inoculate 150 mL of an 16°P wort (17% [wt.vol^-1^] Light spray malt extract, Brewferm, Belgium, supplemented with 0.005 mg.L^-1^ zn^2+^, autoclaved for 10 minutes at 110°C) to a starting concentration of 1.5 X 10^7^ cells.mL^-1^).

The 250 mL bottles were equipped with a water lock and stirring bar after which they were incubated at 14°C, agitated at 150 rpm. Weight loss was measured on a daily basis to track fermentation kinetics. Fermentations were stopped when the daily weight loss was equal or less than 0.05 g. Next, the fermentations were cooled on ice to prevent evaporation of the volatile compounds, and samples for chromatographic analysis (HS-GC-FID, Shimadzu corporation), ethanol (Alcolyzer beer ME, Anton Paar GmbH), sulfite and glycerol (Gallery Plus Beermaster, Thermo Scientific) measurements were taken. The leftover fermented medium was used for sensory analysis.

### Data analysis and representation

All data analyses and visualization were performed in R [48]. Statistical analyses were conducted within the multicomp package (version 1.4–8 [49]). Figures were generated using the ggplot2 package (version 2.2.1 [50]).

## Results

Our goal was to develop and test a CRISPR-based genome editing strategy aimed at modifying the POF phenotype of industrial yeasts, including polyploid and aneuploid yeasts as well as interspecific hybrids. More specifically, we aimed at introducing a loss-of-function SNP mutation in the *FDC1* gene that occurs naturally in many domesticated industrial ale beer yeasts [42].

### Identification of a CRISPR target region to modify the POF phenotype

The first step in CRISPR-based gene editing is finding an appropriate target region and designing the necessary sgRNA sequence [35]. This region needs to meet certain basic requirements. First, the region should harbor a loss-of-function mutation present in the natural biodiversity of *Saccharomyces* yeasts. Introducing such mutation in the same species or a closely related one is regarded as cisgenic modification, which will favor its industrial applicability. Second, the region should be highly conserved between different strains and even species, ensuring that the strategy works in several yeast strains, species and hybrids. Third, the region should contain a neighboring PAM sequence, essential for the correct identification by the Cas9 endonuclease of its target site. Fourth, the region should also be unique in the genome to avoid off-target activity.

**Fig 1:**
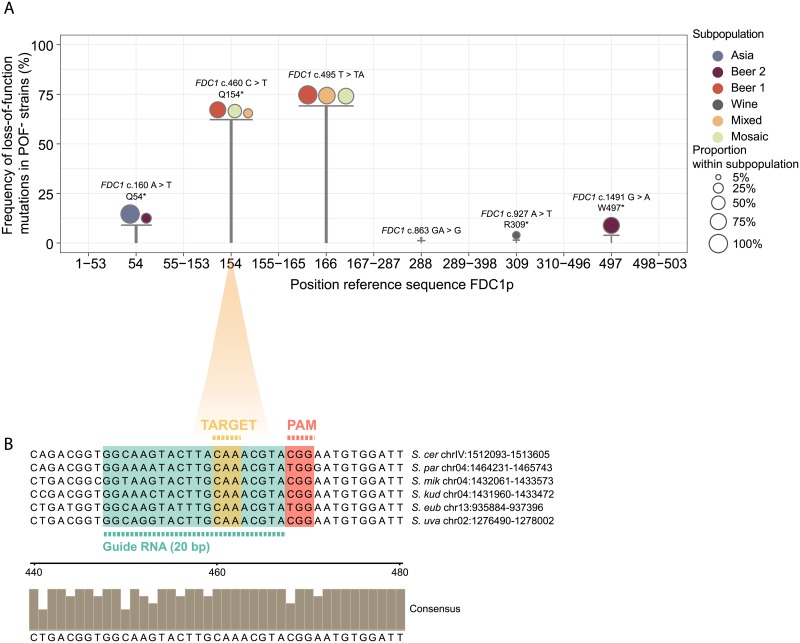
Natural loss-of-function mutations identified in the *FDC1* gene and selection of target mutation. (A) Occurrence of natural loss-of-function mutations in the *FDC1* gene across a collection of 76 POF^-^ *S*. *cerevisiae* strains [33, 42]. Bars indicate the presence and the position of the mutation in Fdc1p based on *S*. *cerevisiae* s288C reference sequence. Height of the bars indicate the frequency of the mutation across the full collection of POF^-^ *S*. *cerevisiae* strains considered. Distribution of the mutation is further dissected across *S*. *cerevisiae* subpopulations (circles—colors) and its proportion within each subpopulation is summarized (circle—size) (exact frequencies are reported in S1 Table). Type and position of the mutation in the coding sequence are annotated on top of each bar. Only POF^-^ *S*. *cerevisiae* strains that harbor homozygous loss-of-function mutations were included in the analysis. (B) Natural *FDC1* loss-of-function point mutation selected for the CRISPR-Cas9 gene editing procedure (yellow cone). The alignment represents a zoom-in of the targeted region in the coding sequence of *FDC1* across six *Saccharomyces* species (-20nt, +20nt from the targeted point mutation). Colored boxes highlight specific areas of the targeted region: the targeted glutamine codon (CAA) that will be replaced by a stop codon (TAA) (yellow box), the PAM sequence (red box) and the guide RNA (green box). The bar-chart represents the alignment of consensus annotation for each position in the targeted region as calculated by Jalview [52].

Analysis of the genome sequences of industrial POF^-^ *S*. *cerevisiae* yeasts, as described by Gallone and coworkers [42], indicates that the disruption of either *PAD1* or *FDC1*, inhibits POF production [28, 33]. The majority (73.80%) of POF^-^ strains from the ‘Beer 1’ lineage [42] share a C to T mutation at position 460 of the *FDC1* coding sequence, that replaces a glutamine residue (CAA) by a stop codon (UAA) (Fig 1A). This premature stop codon likely disrupts the protein function, as both the dimerization domain and the catalytic pocket of the protein are not formed anymore (S3 Fig) [51]. Importantly, for the other species within the *Saccharomyces* species complex with a known *FDC1* sequence, the same C to T mutation at position 460 of the *FDC1* coding sequence introduces a similar stop codon (UAA) (Fig 1B).

Moreover, there is a high degree of conservation in the DNA sequence surrounding this mutation in all 156 previously sequenced *S*. *cerevisiae* strains [42]. Indeed, the sequence of the proposed sgRNA sequence is 100% identical for 155 out of the 156 sequenced *S*. *cerevisiae* strains. In addition, a PAM sequence (NGG) could be found in the close proximity of this mutation (nine nucleotides downstream of the mutation), which is crucial for the correct guidance of the CaCas9 endonuclease to its target (Fig 1B) [45]. Importantly, the same PAM sequence, situated nine nucleotides downstream of the target mutation site, can be found in the *FDC1* sequence of all other sequenced members of the *Saccharomyces* species complex, allowing the design of a possible sgRNA sequence for these *Saccharomyces* species (Fig 1B).

Lastly, possible off-target reactions of the CRISPR system were assessed by blasting the newly designed species-specific 20 nucleotide guide sequences plus possible PAM sequence (NGG) against the genome of the 156 previously sequenced *S*. *cerevisiae* yeasts [42], as well as against the de-novo assembly of the *S*. *eubayanus* genome [53].This analysis shows that the proposed sgRNA sequences are species- and target unique as no other sequences with more than 85% similarity were detected. Moreover, mismatches or gaps in off-target sequences with the highest similarity occur in the first 13 bp immediately upstream of the PAM sequence, which has been shown to be sufficient to achieve a 100% off-target free gene editing in *Saccharomyces* yeasts [54].

Together, this indicates that the proposed region (Fig 1) is an appropriate candidate for effective cisgenic CRISPR-based engineering of the POF phenotype in pure and hybrid *Saccharomyces* species.

### Evaluation of CRISPR efficiency in euploid *S*. *cerevisiae* and non-*cerevisiae* yeasts

Gene editing of industrial *Saccharomyces* yeasts is complicated by two main factors. First, many industrial yeasts are poly- and/or aneuploid, and therefore can contain multiple alleles of the target genes which all need to be modified by the CRISPR system. Analysis of the genomes of industrial ale yeasts shows that these strains have an average ploidy level of 3.52, with some yeasts showing a ploidy level above 4. As POF production is a dominant trait, all alleles of *FDC1* or *PAD1* need to be deactivated to affect the phenotype. Furthermore, CRISPR protocols are generally optimized for lab strains of *S*. *cerevisiae*, and their efficiency for editing non-*cerevisiae* or mixed genomes can be low [35, 46, 54].

To assess the efficiency of CRISPR-based gene editing in polyploid genomes, we introduced the desired nonsense mutation in *FDC1* of different POF^+^ *S*. *cerevisiae* strains with different ploidy levels. Besides a lab strain (haploid and diploid S288c), POF^+^ industrial beer yeasts BE014 and BE020 (diploid) and BE002 and BE074 (triploid) were subjected to the CRISPR transformation. These strains contain two (BE014, BE020) or three (BE002, BE074) functional copies of the *FDC1* gene, and at least one functional *PAD1* gene [42].

Overall, a decrease in the efficiency of CRISPR gene editing was observed with increasing ploidy levels and genome complexity. The haploid and diploid strains showed a high success rate similar to that of the laboratory strains. Introduction of the mutations was observed in 100% (8/8) and 87.5% (7/8) of the investigated colonies for the haploid and diploid variant, respectively. In both diploid beer yeasts, the introduced mutation was homozygous in 100% (10/10 and 5/5 respectively) of the tested colonies. Efficiency in BE074 and BE002 was lower, with homozygous mutations observed in 50%(1/2) and 10% (1/10) of the tested colonies, respectively.

To evaluate the efficiency of the strategy in non-*cerevisiae* species, we introduced the same C to T mutation in the *FDC1* gene of two POF^+^ diploid *S*. *eubayanus* yeasts (WL022 and WL024) (Fig 1). This gene editing was highly successful, with an efficiency of 100% (3/3 and 1/1 respectively) for both strains, yielding POF^-^ variants that may be suitable for industrial application [8, 32].

**Fig 2:**
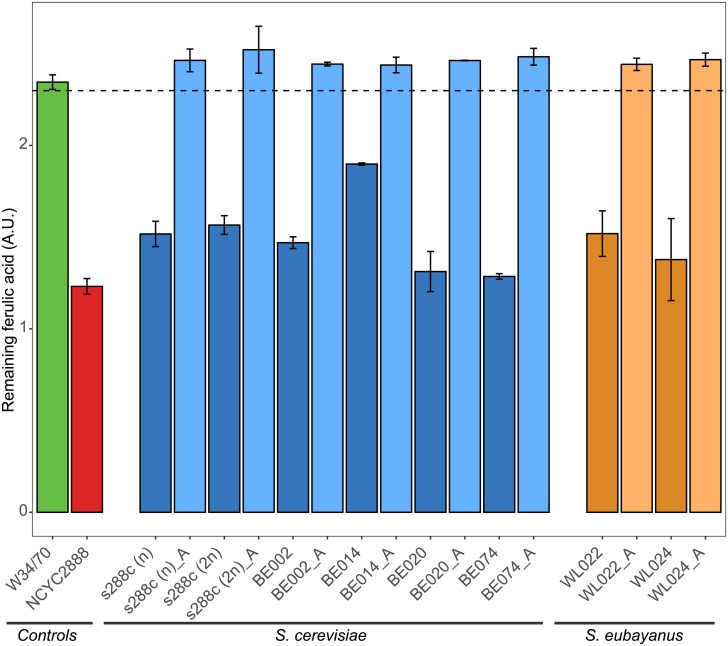
CRISPR mutants lost the ability to convert ferulic acid to 4VG. Yeast cultures were incubated with an excess of ferulic acid, the precursor for the POF aroma. A decrease in ferulic acid indicates POF aroma formation. Remaining ferulic acid was measured for two control strains (POF^-^ control (green); POF^+^ control (red)), six different *S*. *cerevisiae* strains (dark blue) and a gene-edited variant of each (light blue), as well as two *S*. *eubayanus* yeasts (orange) and a gene edited variant of each (light orange). Error bars represent the standard deviation of two biological replicates. The dotted line represents the applied cut-off value (lower border of the 90% confidence interval of the POF^-^control).

Evaluation of the newly formed mutants revealed that the introduction of the homozygous nonsense mutation effectively abolish the ability to produce the unwanted POF aroma (Fig 2).

### Gene editing of interspecific hybrid yeasts can induce loss of chromosomal fragments

Next, the CRISPR-based gene editing was evaluated in interspecific hybrids. Therefore, we first generated POF^+^ hybrids between *S*. *cerevisiae* and *S*. *eubayanus*, after which we tried to remove their POF production by introducing a nonsense mutation in their *FDC1* genes using the developed CRISPR strategy.

Initially, the standard CRISPR-based gene editing strategy [46] was used to introduce the selected SNP mutation in the *S*. *eubayanus* derived *FDC1* allele of the novel generated interspecific hybrids H1 and H2. The *S*. *cerevisiae* parent of H1 (BE011) is POF^-^ and its *FDC1* carries a homozygous nonsense mutation in *FDC1* p.W497* [42]. The *S*. *cerevisiae* parent of H2 (SP003) is also POF^-^ and its *FDC1* is heterozygous for the nonsense mutation p.Q154* and homozygous for the insertion T>TA at position p.166 [42]. As these mutations differ from the one targeted in the developed CRISPR-based gene editing strategy, it allows to determine the species-specificity of the designed strategy towards the alleles derived from their *S*. *eubayanus* and *S*. *cerevisiae* parents.

**Fig 3:**
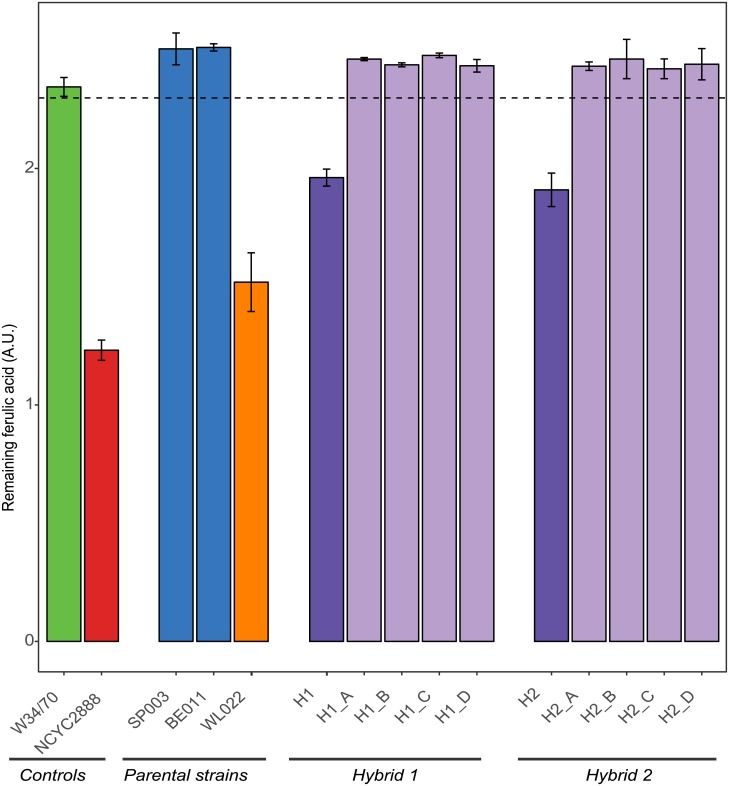
CRISPR mutants lose their ability to convert ferulic acid to 4VG. Yeast cultures were incubated with an excess of ferulic acid, the precursor for the POF aroma. A decrease in ferulic acid indicates POF aroma formation. Remaining ferulic acid was measured for two control strains (POF^-^ control (green); POF^+^ control (red)), *S*. *cerevisiae* and *S*. *eubayanus* parental strains (dark blue and orange, respectively), Interspecific hybrid H1 and H2 (dark purple) with four of their respective CRISPR variants (light purple). Error bars represent the standard deviation of two biological replicates. The dotted line represents the applied cut off value (lower border of the 90% confidence interval of the POF^-^control).

For the first interspecific hybrid, H1, 30 POF^-^ variants were obtained out of 32 tested (94%). Similarly, H2 yielded 21 POF^-^ variants out of 24 (87.5%) (S1 Fig). However, control PCR reaction (primer pair SS_FWSE and SS_RVSE (S10 Table)), which amplifies 490 bp surrounding the DSB induced in the *S*. *eubayanus* derived *FDC1* gene, did not yield an amplification product. Interestingly, further genetic characterization of this region revealed that the CRISPR editing induced the loss of the region downstream of the targeted region in the *S*. *eubayanus* Chr13 (S2 Fig). Genomes of artificial interspecific hybrids are notoriously unstable, and introduction of a double-stranded break likely caused partial loss of the respective chromosome [9, 55, 56]. Whereas this genomic rearrangement yields POF^-^ variants, it is undesirable as it might have major unwanted pleiotropic consequences for other phenotypes. Therefore, the strategy was further optimized to eliminate this detrimental effect in interspecific hybrids. Increasing the concentration of repair template from 5 to 25 μg during the transformation stage and shortening the selection step for the presence of the CRISPR plasmid from two rounds to only one round of selection, allowed us to overcome this interspecific hybrid genome instability. This strategy yielded POF^-^ variants (Fig 3), with a 12.9 (H1, 4/31) and 6.84% (H2; 8/117) efficiency. None of these variants showed partial chromosome loss. Moreover, none of these variants acquired the targeted mutation in the *S*. *cerevisiae FDC1* allele (confirmed via Sanger sequencing), showing the specificity of the designed CRISPR-based genome editing strategy.

### Gene edited variants do not show phenotypic side effects

To determine whether the gene editing procedure introduced any unwanted side effects to the fermentation performance of the yeasts, we evaluated their performance in lab-scale beer fermentations and compared the profiles to those of their respective wild types (WT).

**Fig 4:**
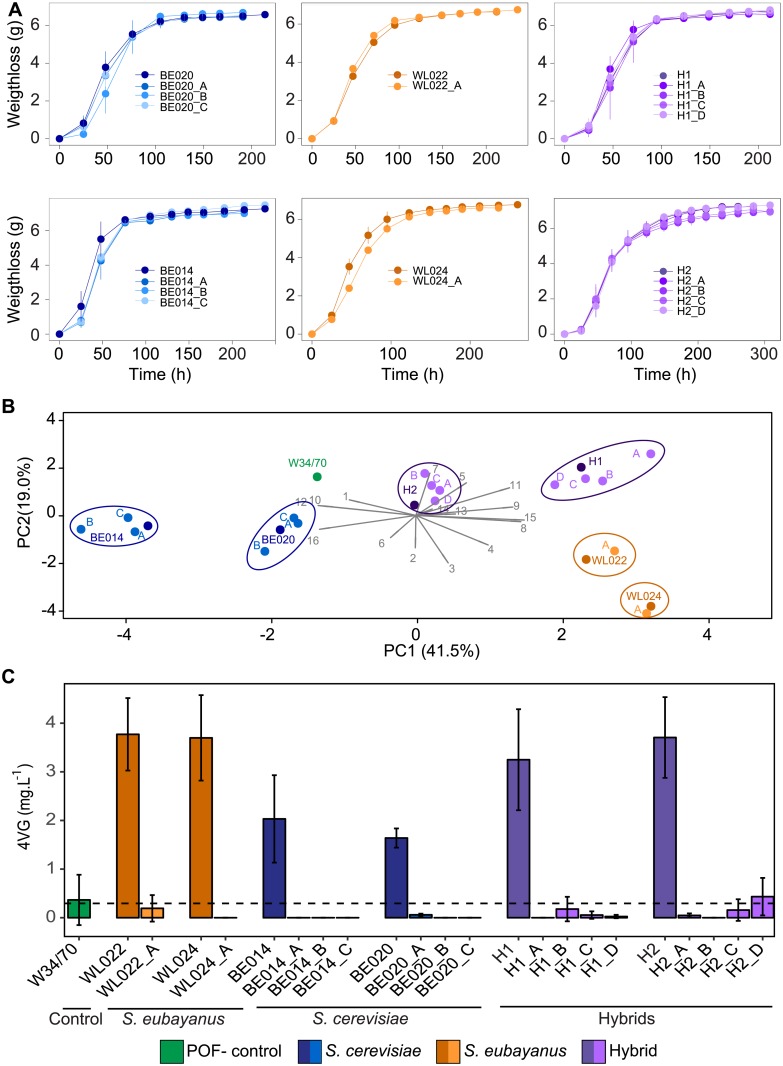
Gene edited variants behave similar to their parental strains in lab scale beer fermentations, except for their POF phenotype. (A), fermentation profiles of BE020, BE014, WL022, WL024, H1 and H2 with their respective gene edited mutants. (B) Principle Component Analysis (PCA) based on the production of 16 different metabolites, describing 60.5% of the total variability. Eigenvectors of the different variables are depicted with numbers ranging from one to 16. 1 = Ethanol, 2 = glycerol, 3 = SO2, 4 = acetaldehyde, 5 = ethyl acetate, 6 = ethyl propionate, 7 = propyl acetate, 8 = isoamyl alcohol, 9 = isobutyl acetate, 10 = ethyl butyrate, 11 = isoamyl acetate, 12 = ethyl hexanoate, 13 = phenetyl alcohol, 14 = ethyl octanoate, 15 = phenetyl acetate and 16 = ethyl decanoate. (C) 4VG production (measured via HS-GC-FID; mg.L ^-1^) of W34/70 (green), *S*. *eubayanus* WL022 and WL024 (dark orange) with their respective gene edited variants (light orange), *S*. *cerevisiae* BE014 and BE020 (dark blue) with their respective gene edited variants (light blue), as well as for artificial interspecific hybrids H1 and H2 (dark purple) with their gene edited variants (light purple). Error bars represent the standard deviation of two biological replicates. Dotted line represents the flavor threshold of 4VG in beer (0.3mg.L^-1^), as reported in [29].

Overall, profiles of the various gene edited variants were similar to those of their WT ancestral strains, with no significant differences in CO_2_ production throughout the fermentation (Fig 4A and S2 Table), with the exception of strain WL022, where the mutant showed a faster fermentation at T2 (Anova-test; P-value <0.001) and T3 (P-value <0.01)). Furthermore, no significant differences were measured in ethanol production at the final stage of the fermentation (S3–S8 Tables).

At the end of the fermentation, 17 different flavor-active metabolites were evaluated. Apart from the targeted phenotype, namely the production of 4VG (see further), no differences between gene edited variants and the WT were observed (Anova and post-hoc Tukey test; P-value > 0.05; S3–S8 Tables), except for *S*. *eubayanus* WL024, where a significant difference was measured for one aroma compound, isoamyl alcohol (P-value = 0.004; average isoamyl alcohol production of WL024 is 227.34 mg.L^-1^ and 253.85 mg.L^-1^ for WL024_A). Principal component analysis (PCA), based on the production of the remaining 16 metabolites, shows a clear clustering of the different gene-edited variants with their respective WT ancestors. This further confirms that apart from the production of POF aroma, the overall flavor profile of the strains remained unchanged compared to that of the parental WT strains (Fig 4B).

As expected, all mutants showed a dramatic decrease in 4VG production (Fig 4C, P-values ranging from <0.001 to 0.033; S3–S8 Tables), with the 4VG concentrations remaining below or around the reported flavor threshold of 0.3 mg.L^-1^ [29].

Subsequent sensorial analysis by a trained panel supported the above-mentioned measurements. Whereas BE014 and BE020 clearly produced clove like off-flavors, the fermentation products obtained with the respective gene edited variants were described as ‘very fruity’ and ‘neutral’. Similarly, mutants of *S*.*eubayanus* strains WL022 and WL024 were described as ‘slightly fruity’ to ‘fruity’. H1’s aroma was defined as being ‘slightly fruity’ with ‘phenolic’ notes. All four gene edited variants of H1 were scored similar by the panel as being ‘very fruity’, highlighting the masking effect of 4VG on fruitiness. A similar trend was observed for H2. Interestingly, the aromatic contribution of all generated gene edited variants was still remarkably different compared to the aroma produced by the reference lager yeast W34/70, revealing the potential of these hybrids to broaden the aromatic diversity of lager beers, without introducing unwanted clove like phenolic off-flavors.

## Discussion

Today’s beer market is characterized by an increased demand for niche products and diversification [57]. These market forces have led to an increased interest into novel beer yeasts that can impart new aromas. However, some of the most interesting strains are characterized by the production of the undesirable aroma compound 4VG. Here we describe a new CRISPR-based gene editing strategy that allows to specifically modify 4VG production in various yeasts, including feral *S*. *cerevisiae* isolates, non-*Saccharomyces* strains and interspecific hybrids.

Compared to previous approaches aimed at modifying industrially-relevant phenotypes in yeast [36, 38, 54], this study introduces a naturally-occurring point mutation, rather than deleting the gene of interest, thereby generating cisgenic variants that are likely exempted from GM regulations within countries like Argentina, USA, Japan and Brazil [40, 41, 58].

Gene editing efficiency ranged from 100% for lab strains and diploid industrial *S*. *cerevisiae* strains to 10% for more complex, poly- and euploid industrial *S*. *cerevisiae* yeasts. We also report the first CIRPSR-based engineering of *S*. *eubayanus*, which was also highly efficient (100%). The reported gene editing efficiencies are in line with previous reported efficiencies in industrial relevant *S*. *cerevisiae* strains when performing CRISPR-based gene deletions (1.3% up to 100% [36, 54, 59]).

Although CRISPR-based editing was previously reported to be an efficient route to modify *S*. *pastorianus* [38], the Cas9-induced double-stranded DNA break caused an unexpected and undesirable partial chromosome loss [35]. Off-target activity of CRISPR genome editing in haploid or homozygous *Saccharomyces* yeasts has been shown to be very rare [59]. More recently however, allele specific gene editing in artificial *S*. *cerevisiae* X *S*. *eubayanus* hybrids was reported to cause loss of heterozygosity (LOH). Specifically, the induced double-stranded break in the *S*. *eubayanus* derived locus was not repaired by the provided repair template via homology-directed repair, but rather repaired via LOH [60]. Although further research is needed, the fact that *FDC1* is located in the subtelomeric region in *S*. *cerevisiae* (Chr IV) and *S*. *euybayanus* (Chr13) could favor partial chromosome loss over repair via LOH. One way to reduce such unwanted structural rearrangements is to design the repair template in such a way that the PAM site is inactivated [54]. This prevents continued cutting of the site by the Cas9 endonuclease after successful introduction of the desired mutation [61]. However, this strategy is not ideal for editing organisms that are targeted for food production, as in most cases the resulting mutant would not be cisgenic and thus subjected to GM laws. We therefore modified the gene editing protocol by increasing the amount of repair template and reducing the CRISPR-Cas9 endonuclease activity, which seemed to reduce the unwanted genomic rearrangements and increased repair via HDR. The proposed technique showed a seven to 12% efficiency in specifically introducing a single SNP in the subtelomeric *S*. *eubayanus* derived *FDC1* gene in novel generated interspecific hybrids.

Overall, some of the generated POF^-^ variants show great potential for industrial application, as their unique aroma profiles are no longer masked by 4VG. Additionally, the CRISPR gene editing strategy described in our study offers a general tool for tuning the characteristics of various aneuploid and non-*Saccharomyces* yeasts. Specifically, the combination of our gene editing protocol with the ever-increasing number of identified quantitative trait loci (QTL) represents a formidable opportunity to obtain superior industrial yeasts through gene editing [62].

## Supporting information

S1 Fig POF phenotype CRISPR variants with standard protocol. Remaining ferulic acid was measured for two control strains (POF^-^ control (green); POF^+^ control (red)), Interspecific hybrid one and two (dark green) with their respective POF^-^ (light green) and POF^+^ (dark red) CRISPR variants, obtained via the standard CRISPR protocol. Error bars represent the standard deviation of two biological replicates. Dotted line represents the used cut off value (lower border of the 90% confidence interval of the POF^-^control).
(EPS)

S2 Fig Standard CRISPR protocol causes genetic instability in complex hybrid genomes. Three primer pairs were designed, each targeting a different part of the *S*. *eubayanus* derived Chr13 in the generated interspecific hybrids H1 and H2. Primer pair one and two amplify a region 4kb and 8b upstream of the introduced DBS respectively. Primer pair three amplifies a region 1kb downstream of the introduced DBS on the *S*. *eubayanus* derived chromosome of our hybrids (visual representation not depicted to scale). Primer pair three does not yield any of the expected sized PCR product for gene edited variants generated with the standard CRISPR protocol (H1_1 and H2_1) but does for gene edited variants generated with the adjusted CRISPR protocol (H1_A and H2_A), proposed within this manuscript.
(EPS)

S3 Fig 3D crystal structure of Fdc1p. Fdc1p acts as a dimer (blue and red form one monomer, cyan and orange, the second one).Blue and cyan parts of the molecules represent the N-terminal part of the protein that it still formed before the stop-gain mutation p.Q154, whilst the red and orange parts disappears when Q154 is replaced by a stop codon [51]. Two 4VG molecules, which are bound to the catalytic pockets of both monomers, are colored in green.
(EPS)

S4 Fig Hybrid confirmation through species specific multiplex PCR. Two primer pairs were used for the species-specific multiplex PCR, each targeting a specific part of one of the parental species’ genome. Primers Scer F2 (5’-GCG CTT TAC ATT CAG ATC CCG AG-3’) and Scer R2 (5’-TAA GTT GGT TGT CAG CAA GAT TG-3’) amplify a 150-bp amplicon of the *S*. *cerevisiae* genome [63]. Primers Seub F3 (5’-GTC CCT GTA CCA ATT TAA TAT TGC GC-3’) and Seub R2 (5’-TTT CAC ATC TCT TAG TCT TTT CCA GAC G-3’) generate a 228-bp *S*. *eubayanus*-specific amplicon The PCR conditions were as follows: 3 min at 95°C, 30 cycles of 30 s at 95°C, 30 s at 58°C, and 30 s of 72°C, followed by a final cycle of 5 min at 72°C and subsequent cooling to room temperature (RT). Gel electrophoreses shows both the *cerevisiae* and *eubayanus* specific PCR product for hybrids H1 and H2, as well as for *S*. *pastorianus* W34/70. The *S*. *cerevisiae* control strains WI009, BE001 and SP003 only show the *S*. *cerevisiae* specific PCR product, whereas *S*. *eubayanus* WL022 only shows the *S*. *eubayanus* specific PCR product.
(TIF)

S1 Table Overview occurrence of natural loss-of-function mutations in the FDC1 gene across a collection of 76 POF^-^ *S*. *cerevisiae* strains. Total frequency is the frequency of the mutations across the complete collection of POF^-^ *S*. *cerevisiae* strain collection. Column four to nine show the frequency of each mutation across *S*. *cerevisiae* sub-populations, as described earlier [44]. Only POF^-^ *S*. *cerevisiae* strains that harbor homozygous loss-of-function mutations were included in the analysis.
(PDF)

S2 Table Statistical analysis of the weight loss measured during fermentation between gene edited variants and their respective WT (from time point 1 to end of the fermentation). P-values were obtained using ANOVA. All statistical analysis were conducted in R, with the multcomp package (* P-value < 0.05; ** P-value <0.01; *** P-values <0.001). “/” means fermentations were stopped before this time point.
(PDF)

S3 Table Statistical analysis of the phenotypic behavior of H1 compared to the H1 gene-edited variants. Column two represents the P-values obtained with ANOVA. Column three to twelve represent the obtained P-values of a post-hoc Tukey test. All statistical analyses were conducted in R, within the multcomp package (* P-value < 0.05; ** P-value <0.01; *** P-values <0.001).
(PDF)

S4 Table Statistical analysis of the phenotypic behavior of H2 compared to the H2 gene-edited variants. Column two represents the P-values obtained with ANOVA. Column three to twelve represent the obtained P-values of a post-hoc Tukey test. All statistical analyses were conducted in R, within the multcomp package (* P-value < 0.05; ** P-value <0.01; *** P-values <0.001).
(PDF)

S5 Table Statistical analysis of the phenotypic behavior of BE014 compared to its gene-edited variants. Column two represents the P-values obtained with ANOVA. Column three to twelve represent the obtained P-values of a post-hoc Tukey test. All statistical analyses were conducted in R, within the multcomp package (* P-value < 0.05; ** P-value <0.01; *** P-values <0.001).
(PDF)

S6 Table Statistical analysis of the phenotypic behavior of BE020 compared to its gene-edited variants. Column two represents the P-values obtained with ANOVA. Column three to twelve represent the obtained P-values of a post-hoc Tukey test. All statistical analyses were conducted in R, within the multcomp package (* P-value < 0.05; ** P-value <0.01; *** P-values <0.001).
(PDF)

S7 Table Statistical analysis of the phenotypic behavior of WL022 compared to its gene-edited variant. Column two represents the P-values obtained with ANOVA. Column three to twelve represent the obtained P-values of a post-hoc Tukey test. All statistical analyses were conducted in R, within the multcomp package (* P-value < 0.05; ** P-value <0.01; *** P-values <0.001).
(PDF)

S8 Table Statistical analysis of the phenotypic behavior of WL024 compared to its gene-edited variant. Column two represents the P-values obtained with ANOVA. Column three to twelve represent the obtained P-values of a post-hoc Tukey test. All statistical analyses were conducted in R, within the multcomp package (* P-value < 0.05; ** P-value <0.01; *** P-values <0.001).
(PDF)

S9 Table Overview aroma and ethanol production from lab scale lager beer fermentation tests. Quantified yeast-related aroma compounds are represented as concentrations (mg.L^-1^), total weight loss as grams (g), ethanol production as volume percentage. Glycerol and SO_2_ production are represented as concentrations (g.L^-1^ and mg.L^-1^ respectively). H_2_S production capacity is qualitatively indicated (+,+-, -). Lastly, the used score legend for flavors during sensory analysis was: VS = very slightly; S = slightly; V = very; N = neutral; FR = fruity; POF = cloves, phenolic; FRESH = fresh.
(PDF)

S10 Table Overview used primers. (PDF)

## References

[1] González SS, Barrio E, Gafner J, Querol A. Natural hybrids from *Saccharomyces cerevisiae*, *Saccharomyces bayanus* and *Saccharomyces kudriavzevii* in wine fermentations. FEMS Yeast Res. 2006; 6(8):1221–34. 10.1111/j.1567-1364.2006.00126.x 17156019

[2] Borneman AR, Desany BA, Riches D, Affourtit JP, Forgan AH, Pretorius IS, et al The genome sequence of the wine yeast VIN7 reveals an allotriploid hybrid genome with *Saccharomyces cerevisiae* and *Saccharomyces kudriavzevii* origins. FEMS Yeast Res. 2012; 12(1):88–96. 10.1111/j.1567-1364.2011.00773.x 22136070

[3] Rainieri S, Zambonelli C, Kaneko Y. *Saccharomyces sensu stricto*: systematics, genetic diversity and evolution. J Biosci Bioeng. 2003; 96(1):1–9. 16233475

[4] Boynton PJ, Greig D. The ecology and evolution of non-domesticated *Saccharomyces* species. Yeast. 2014; 31(12):449–62. 10.1002/yea.3040 25242436 PMC4282469

[5] Dujon BA, Louis EJ. Genome diversity and evolution in the budding yeasts (*Saccharomycotina*). Genetics. 2017; 206(2):717–50. 10.1534/genetics.116.199216 28592505 PMC5499181

[6] Gibson B, Liti G. *Saccharomyces pastorianus*: genomic insights inspiring innovation for industry. Yeast. 2014; 28(28):17–27. 10.1002/yea.3033 25088523

[7] Libkind D, Hittinger CT, Valério E, Gonçalves C, Dover J, Johnston M, et al Microbe domestication and the identification of the wild genetic stock of lager-brewing yeast. Proc Natl Acad Sci U S A. 2011; 108:14539–44. 10.1073/pnas.1105430108 21873232 PMC3167505

[8] Gibson B, Geertman J-MA, Hittinger CT, Krogerus K, Libkind D, Louis EJ, et al New yeasts—new brews: modern approaches to brewing yeast design and development. FEMS Yeast Res. 2017; 1–32. 10.1093/femsyr/fox038 28582493

[9] Dunn B, Sherlock G. Reconstruction of the genome origins and evolution of the hybrid lager yeast *Saccharomyces pastorianus*. Genome Res. 2008; 18:1610–23. 10.1101/gr.076075.108 18787083 PMC2556262

[10] Querol A, Bond U. The complex and dynamic genomes of industrial yeasts. FEMS Microbiol Lett. 2009; 293(1):1–10. 10.1111/j.1574-6968.2008.01480.x 19175410

[11] Christine LJ, Marc L, Catherine D, Claude E, Jean-Luc L, Michel A, et al Characterization of natural hybrids of *Saccharomyces cerevisiae* and *Saccharomyces bayanus var*. *uvarum*. FEMS Yeast Res. 2007; 7(4):540–9. 10.1111/j.1567-1364.2007.00207.x 17302940

[12] González SS, Barrio E, Querol A. Molecular characterization of new natural hybrids of *Saccharomyces cerevisiae* and *S*. *kudriavzevii* in brewing. Appl Environ Microbiol. 2008; 74(8):2314–20. 10.1128/AEM.01867-07 18296532 PMC2293171

[13] Krogerus K, Preiss R, Gibson B. A Unique *Saccharomyces cerevisiae* × *Saccharomyces uvarum* Hybrid Isolated From Norwegian Farmhouse Beer: Characterization and Reconstruction. Front Microbiol. 2018; 9. 10.3389/fmicb.2018.02253 PMC6165869 30319573

[14] Steensels J, Snoek T, Meersman E, Nicolino MP, Voordeckers K, Verstrepen KJ. Improving industrial yeast strains: exploiting natural and artificial diversity. FEMS Microbiol Rev. 2014; 1–49. 24724938 10.1111/1574-6976.12073 PMC4293462

[15] Snoek T, Picca Nicolino M, Van Den Bremt S, Mertens S, Saels V, Verplaetse A, et al Large-scale robot-assisted genome shuffling yields industrial *Saccharomyces cerevisiae* yeasts with increased ethanol tolerance. Biotechnol Biofuels. 2015; 8(1). 10.1186/s13068-015-0216-0 PMC4354739 25759747

[16] Steensels J, Meersman E, Snoek T, Saels V, Verstrepen KJ. Large-scale selection and breeding to generate industrial yeasts with superior aroma production. Appl Environ Microbiol. 2014; 80(22):6965–75. 10.1128/AEM.02235-14 25192996 PMC4249010

[17] Serra A, Strehaiano P, Taillandier P. Influence of temperature and pH on *Saccharomyces bayanus var*. *uvarum* growth; impact of a wine yeast interspecific hybridization on these parameters. Int J Food Microbiol. 2005; 104:257–65. 10.1016/j.ijfoodmicro.2005.03.006 15979182

[18] Sebastiani F, Barberio C, Casalone E, Cavalieri D, Polsinelli M. Crosses between *Saccharomyces cerevisiae* and *Saccharomyces bayanus* generate fertile hybrids. Res Microbiol. 2002; 153:53–8. 11881899 10.1016/s0923-2508(01)01286-4

[19] Nikulin J, Krogerus K, Gibson B. Alternative *Saccharomyces* interspecies hybrid combinations and their potential for low-temperature wort fermentation. Yeast. 2018; 35(1):113–27. 10.1002/yea.3246 28755430 PMC5811906

[20] Krogerus K, Seppänen-Laakso T, Castillo S, Gibson B. Inheritance of brewing-relevant phenotypes in constructed *Saccharomyces cerevisiae* x *Saccharomyces eubayanus* hybrids. Microb Cell Fact. BioMed Central; 2017; 16(1):66. 10.1186/s12934-017-0679-8 PMC5399851 28431563

[21] Krogerus K, Magalhães F, Vidgren V, Gibson B. New lager yeast strains generated by interspecific hybridization. J Ind Microbiol Biotechnol. 2015; 42(42):769–78. 25682107 10.1007/s10295-015-1597-6 PMC4412690

[22] Magalhães F, Krogerus K, Vidgren V, Sandell M, Gibson B. Improved cider fermentation performance and quality with newly generated *Saccharomyces cerevisiae* × *Saccharomyces eubayanus* hybrids. J Ind Microbiol Biotechnol. Springer Berlin Heidelberg; 2017; 44(8):1–11. 10.1007/s10295-017-1947-7 PMC5511608 28451838

[23] Mertens S, Steensels J, Saels V, De Rouck G, Aerts G, Verstrepen KJ. A large set of newly created interspecific yeast hybrids increases aromatic diversity in lager beers. Appl Environ Microbiol. 2015; 81(23). 10.1128/AEM.02464-15 PMC4651086 26407881

[24] Hebly M, Brickwedde A, Bolat I, Driessen MRM, de Hulster E a. F, van den Broek M, et al *S*. *cerevisiae* x *S*. *eubayanus* interspecific hybrid, the best of both worlds and beyond. FEMS Yeast Res. 2015; 15(3):fov005. 10.1093/femsyr/fov005 25743788

[25] Arroyo-López FN, Orlić S, Querol A, Barrio E. Effects of temperature, pH and sugar concentration on the growth parameters of *Saccharomyces cerevisiae*, *S*. *kudriavzevii* and their interspecific hybrid. Int J Food Microbiol. 2009; 131:120–7. 10.1016/j.ijfoodmicro.2009.01.035 19246112

[26] Bellon JR, Eglinton JM, Siebert TE, Pollnitz AP, Rose L, De Barros Lopes M, et al Newly generated interspecific wine yeast hybrids introduce flavour and aroma diversity to wines. Appl Microbiol Biotechnol. 2011; 91:603–12. 10.1007/s00253-011-3294-3 21538112

[27] Steensels J, Verstrepen KJ. Taming Wild Yeast: Potential of Conventional and Nonconventional Yeasts in Industrial Fermentations. Annu Rev Microbiol. 2014; 68(April):61–80. 24773331 10.1146/annurev-micro-091213-113025

[28] Mukai N, Masaki K, Fujii T, Iefuji H. Single nucleotide polymorphisms of *PAD1* and *FDC1* show a positive relationship with ferulic acid decarboxylation ability among industrial yeasts used in alcoholic beverage production. J Biosci Bioeng. 2014; 118(1):50–5. 10.1016/j.jbiosc.2013.12.017 24507903

[29] Vanbeneden N, Gils F, Delvaux F, Delvaux FR. Formation of 4-vinyl and 4-ethyl derivatives from hydroxycinnamic acids: Occurrence of volatile phenolic flavour compounds in beer and distribution of Pad1-activity among brewing yeasts. Food Chem. 2008; 107:221–30.

[30] Maclean CJ, Greig D. Prezygotic reproductive isolation between *Saccharomyces cerevisiae* and *Saccharomyces paradoxus*. BMC Evol Biol. 2008; 8(1). 10.1186/1471-2148-8-1 PMC2249576 18179683

[31] Alexander WG, Peris D, Pfannenstiel BT, Opulente DA, Kuang M, Hittinger CT. Efficient engineering of marker-free synthetic allotetraploids of *Saccharomyces*. Fungal Genet Biol. Elsevier Inc.; 2016; 89:10–7. 10.1016/j.fgb.2015.11.002 PMC4789119 26555931

[32] Diderich JA, Weening SM, van den Broek M, Pronk JT, Daran J-MG. Selection of Pof-*Saccharomyces eubayanus* Variants for the Construction of *S*. *cerevisiae* × *S*. *eubayanus* Hybrids With Reduced 4-Vinyl Guaiacol Formation. Front Microbiol. 2018; 9:1–17. 30100898 10.3389/fmicb.2018.01640 PMC6074607

[33] Mertens S, Steensels J, G. B, V Kevin J. Rapid Screening Method for Phenolic Off-Flavor (POF) Production in Yeast. J Am Soc Brew Chem. 2017; 75(4):318–23.

[34] Sander JD, Joung JK. CRISPR-Cas systems for editing, regulating and targeting genomes. Nature Biotechnology. 2014. 10.1038/nbt.2842 PMC4022601 24584096

[35] Mans R, van Rossum HM, Wijsman M, Backx A, Kuijpers NGA, van den Broek M, et al CRISPR/Cas9: A molecular Swiss army knife for simultaneous introduction of multiple genetic modifications in *Saccharomyces cerevisiae*. FEMS Yeast Res. Oxford University Press; 2015; 15(2):1–15. 10.1093/femsyr/fov004 PMC4399441 25743786

[36] Vigentini I, Gebbia M, Belotti A, Foschino R, Roth FP. CRISPR/Cas9 system as a valuable genome editing tool for wine yeasts with application to decrease urea production. Front Microbiol. Frontiers; 2017; 8:2194. 10.3389/fmicb.2017.02194 PMC5678006 29163459

[37] Denby CM, Li RA, Vu VT, Costello Z, Lin W, Chan LJG, et al Industrial brewing yeast engineered for the production of primary flavor determinants in hopped beer. Nat Commun. Nature Publishing Group; 2018; 9(1):965. 10.1038/s41467-018-03293-x PMC5861129 29559655

[38] de Vries ARG, de Groot PA, van den Broek M, Daran JMG. CRISPR-Cas9 mediated gene deletions in lager yeast *Saccharomyces pastorianus*. Microb Cell Fact. 2017; 16(1):222 10.1186/s12934-017-0835-1 29207996 PMC5718131

[39] Pablo Orozco. Argentina and Brazil merge law and science to regulate new breeding techniques—Alliance for Science. 29 January [Internet]. 2018 [cited 2018 Aug 20]. https://allianceforscience.cornell.edu/blog/2018/01/argentina-and-brazil-merge-law-and-science-to-regulate-new-breeding-techniques/.

[40] Waltz E. Gene-edited CRISPR mushroom escapes US regulation. Nature. 2016; 532(7599):293–293. 10.1038/nature.2016.19754 27111611

[41] Ishii T, Araki M. A future scenario of the global regulatory landscape regarding genome-edited crops. GM Crops Food. 2017; 8(1):44–56. 10.1080/21645698.2016.1261787 27960622 PMC5592978

[42] Gallone B, Steensels J, Prahl T, Soriaga L, Saels V, Herrera-Malaver B, et al Domestication and Divergence of *Saccharomyces cerevisiae* Beer Yeasts. Cell. 2016; 166(6):1397–1410.e16. 10.1016/j.cell.2016.08.020 27610566 PMC5018251

[43] Rodríguez ME, Pérez-Través L, Sangorrín MP, Barrio E, Lopes CA. *Saccharomyces eubayanus* and *Saccharomyces uvarum* associated with the fermentation of *Araucaria araucana* seeds in Patagonia. FEMS Yeast Res. Oxford University Press; 2014; 14(6):948–65. 10.1111/1567-1364.12183 25041507

[44] Baker Brachmann C, Davies A, Cost GJ, Caputo E, Li J, Hieter P, et al Designer deletion strains derived from *Saccharomyces cerevisiae* S288C: A useful set of strains and plasmids for PCR-mediated gene disruption and other applications. Yeast. 1998; 14(2):115–32. 10.1002/(SICI)1097-0061(19980130)14:2<115::AID-YEA204>3.0.CO;2-2 9483801

[45] Vyas VK, Barrasa MI, Fink GR. A Candida albicans CRISPR system permits genetic engineering of essential genes and gene families. Sci Adv. American Association for the Advancement of Science; 2015; 1(3):e1500248–e1500248. 10.1126/sciadv.1500248 PMC4428347 25977940

[46] Vyas VK, Bushkin GG, Bernstein DA, Getz MA, Sewastianik M, Barrasa MI, et al New CRISPR mutagenesis strategies reveal variation in repair mechanisms among fungi. Msph (in Press). 2018; 1–24. 10.1128/mSphere.00154-18 PMC5917429 29695624

[47] Sikorski RS, Hieter P. A system of shuttle vectors and yeast host strains designed for efficient manipulation of DNA in *Saccharomyces cerevisiae*. Genetics. 1989; 122(1):19–27. 2659436 10.1093/genetics/122.1.19 PMC1203683

[48] Team R. R: a language and environment for statistical computing. 2013, posting date [Internet]. [cited 2014 Jan 1]. http://www.r-project.org/.

[49] Hothorn T, Bretz F, Westfall P, Heiberger RM, Schuetzenmeister A, Scheibe S. multcomp: simultaneous inference in general parametric models. R package version 1.4–8. Vienna, Austria; 2013.

[50] Wickham H, Winson C. ggplot2: An implementation of the Grammar of Graphics." R package version 2.2.1. 2008.

[51] Bhuiya MW, Lee SG, Jez JM, Yu O. Structure and mechanism of ferulic acid decarboxylase (*FDC1*) from *saccharomyces cerevisiae*. Appl Environ Microbiol. American Society for Microbiology; 2015; 81(12):4216–23. 10.1128/AEM.00762-15 PMC4524143 25862228

[52] Waterhouse AM, Procter JB, Martin DMA, Clamp M and Barton GJ. Jalview Version 2—a multiple sequence alignment editor and analysis workbench. Bioinformatics. 2009; 25(9) 1189–1191. 10.1093/bioinformatics/btp033 19151095 PMC2672624

[53] Baker E, Wang B, Bellora N, Peris D, Hulfachor AB, Koshalek J et al The Genome Sequence of *Saccharomyces eubayanus* and the Domestication of Lager-Brewing Yeasts. Mol Biol Evol. 2015;32:2818–2831. 10.1093/molbev/msv168 26269586 PMC4651232

[54] Jakočinas T, Bonde I, Herrgård M, Harrison SJ, Kristensen M, Pedersen LE, et al Multiplex metabolic pathway engineering using CRISPR/Cas9 in Saccharomyces cerevisiae. Metab Eng. 2015; 28:213–22. 10.1016/j.ymben.2015.01.008 25638686

[55] Piotrowski JS, Nagarajan S, Kroll E, Stanbery A, Chiotti KE, Kruckeberg AL, et al Different selective pressures lead to different genomic outcomes as newly-formed hybrid yeasts evolve. BMC Evol Biol. BioMed Central Ltd; 2012; 12(1):1–46. 10.1186/1471-2148-12-46 PMC3372441 22471618

[56] Lopandic K. *Saccharomyces* interspecies hybrids as model organisms for studying yeast adaptation to stressful environments. Yeast. Wiley-Blackwell; 2018; 35(1):21–38. 10.1002/yea.3294 29131388

[57] Colen L, Swinnen J. Economic Growth, Globalisation and Beer Consumption. J Agric Econ. Wiley/Blackwell (10.1111); 2016; 67(1):186–207.

[58] Edenbrandt AK, House LA, Gao Z, Olmstead M, Gray D. Consumer acceptance of cisgenic food and the impact of information and status quo. Food Qual Prefer. Elsevier; 2018; 69:44–52.

[59] Stovicek V, Holkenbrink C, Borodina I. CRISPR/Cas system for yeast genome engineering: advances and applications. FEMS Yeast Res. Oxford University Press; 2017; 17(5). 10.1093/femsyr/fox030 PMC5812514 28505256

[60] Vries ARG de, Couwenberg LGF, Broek M van den, la Torre Cortes P de, Horst J ter, Pronk JT, et al. Allele-specific genome editing using CRISPR-Cas9 causes off-target mutations in diploid yeast. bioRxiv. 2018.

[61] DiCarlo JE, Chavez A, Dietz SL, Esvelt KM, Church GM. Safeguarding CRISPR-Cas9 gene drives in yeast. Nat Biotechnol. Nature Publishing Group; 2015; 33(12):1250–5. 10.1038/nbt.3412 PMC4675690 26571100

[62] Liti G, Louis EJ. Advances in Quantitative Trait Analysis in Yeast. PLoS Genet. Public Library of Science; 2012; 8(8):e1002912. 10.1371/journal.pgen.1002912 PMC3420948 22916041

[63] Muir A, Harrison E, Wheals A. A multiplex set of species-specific primers for rapid identification of members of the genus *Saccharomyces*. FEMS Yeast Res. 2011; 11:552–63. 10.1111/j.1567-1364.2011.00745.x 22093682

