# Correction: Reducing phenolic off-flavors through CRISPR-based gene editing of the *FDC1* gene in *Saccharomyces cerevisiae* x *Saccharomyces eubayanus* hybrid lager beer yeasts

**DOI:** 10.1371/journal.pone.0224525

**Published:** 2019-10-24

**Authors:** Stijn Mertens, Brigida Gallone, Jan Steensels, Beatriz Herrera-Malaver, Jeroen Cortebeek, Robbe Nolmans, Veerle Saels, Valmik K. Vyas, Kevin J. Verstrepen

There are errors in the Funding statement. The correct Funding statement is as follows: The research reported in this article was supported by grants to KJV from Vlaams Instituut voor Biotechnologie (VIB), European Molecular Biology Organization Young Investigator Programme (EMBO YIP), and a European Research Council (ERC) Consolidator Grant CoG682009. The funders had no role in study design, data collection and analysis, decision to publish, or preparation of the manuscript.
