# Supplementary figures and images for: Reducing phenolic off-flavors through CRISPR-based gene editing of the FDC1 gene in Saccharomyces cerevisiae x Saccharomyces eubayanus hybrid lager beer yeasts
Source: PLoS One. 2019 Jan 9;14(1):e0209124. doi: 10.1371/journal.pone.0209124 (PMC6326464; doi:10.1371/journal.pone.0209124)

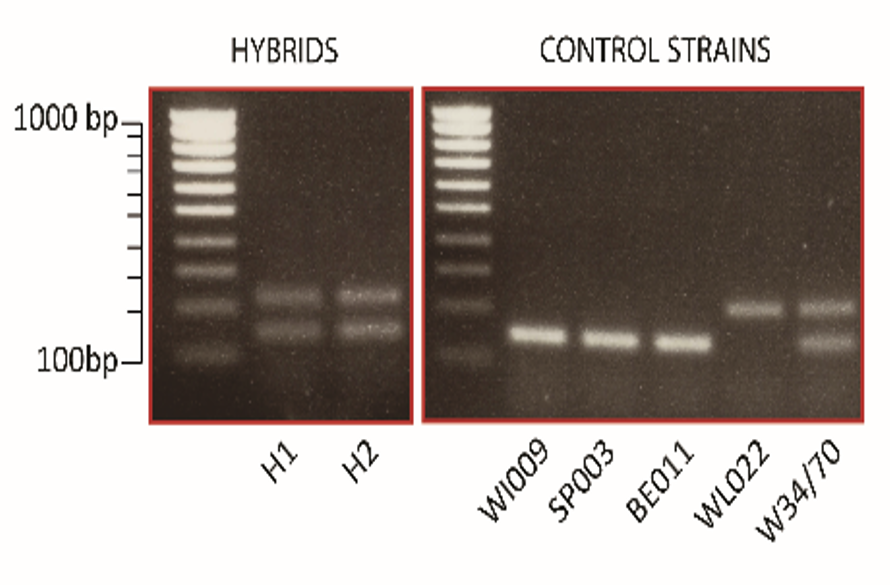

Supplement: S4 Fig — Two primer pairs were used for the species-specific multiplex PCR, each targeting a specific part of one of the parental species’ genome. Primers Scer F2 (5’-GCG CTT TAC ATT CAG ATC CCG AG-3’) and Scer R2 (5’-TAA GTT GGT TGT CAG CAA GAT TG-3’) amplify a 150-bp amplicon of the S. cerevisiae genome [63]. Primers Seub F3 (5’-GTC CCT GTA CCA ATT TAA TAT TGC GC-3’) and Seub R2 (5’-TTT CAC ATC TCT TAG TCT TTT CCA GAC G-3’) generate a 228-bp S. eubayanus-specific amplicon The PCR conditions were as follows: 3 min at 95°C, 30 cycles of 30 s at 95°C, 30 s at 58°C, and 30 s of 72°C, followed by a final cycle of 5 min at 72°C and subsequent cooling to room temperature (RT). Gel electrophoreses shows both the cerevisiae and eubayanus specific PCR product for hybrids H1 and H2, as well as for S. pastorianus W34/70. The S. cerevisiae control strains WI009, BE001 and SP003 only show the S. cerevisiae specific PCR product, whereas S. eubayanus WL022 only shows the S. eubayanus specific PCR product. (TIF) [file pone.0209124.s004.tif]
