# Supplementary material for: Reducing phenolic off-flavors through CRISPR-based gene editing of the FDC1 gene in Saccharomyces cerevisiae x Saccharomyces eubayanus hybrid lager beer yeasts
Source: PLoS One. 2019 Jan 9;14(1):e0209124. doi: 10.1371/journal.pone.0209124 (PMC6326464; doi:10.1371/journal.pone.0209124)
Supplement: S1 Table — Total frequency is the frequency of the mutations across the complete collection of POF- S. cerevisiae strain collection. Column four to nine show the frequency of each mutation across S. cerevisiae sub-populations, as described earlier [44]. Only POF- S. cerevisiae strains that harbor homozygous loss-of-function mutations were included in the analysis. (PDF) [file pone.0209124.s005.pdf]

S1 Table. Overview occurrence of natural loss-of-function mutations in the *FDC1* gene across a collection of 76 POF$^-$ *S. cerevisiae* strains.

		Frequency (%)						
Position	Name	Total frequency	Asia	Beer2	Beer1	Wine	Mixed	Mosaic
54	*FDC1 c.160 A>T (Q54*)*	9.33	100.00	25.00	NA	NA	NA	NA
154	*FDC1 c.460 C>T (Q154*)*	62.67	NA	NA	73.81	NA	20.00	50.00
166	*FDC1 c.495 T>TA*	69.33	NA	NA	97.62	NA	80.00	70.00
288	*FDC1 c.863 GA>G*	1.33	NA	NA	NA	NA	NA	1.00
309	*FDC1 c.927 A>T (R309*)*	1.33	NA	NA	NA	12.50	NA	NA
497	*FDC1 c.1491 G>A (W497*)*	4.00	NA	75.00	NA	NA	NA	NA

Total frequency is the frequency of the mutations across the complete collection of POF$^-$ *S. cerevisiae* strain collection. Column four to nine show the frequency of each mutation across *S. cerevisiae* sub populations, as described earlier [44]. Only POF$^-$ *S. cerevisiae* strains that harbor homozygous loss-of-function mutations were included in the analysis.
