# Supplementary material for: Reducing phenolic off-flavors through CRISPR-based gene editing of the FDC1 gene in Saccharomyces cerevisiae x Saccharomyces eubayanus hybrid lager beer yeasts
Source: PLoS One. 2019 Jan 9;14(1):e0209124. doi: 10.1371/journal.pone.0209124 (PMC6326464; doi:10.1371/journal.pone.0209124)
Supplement: S2 Table — P-values were obtained using ANOVA. All statistical analysis were conducted in R, with the multcomp package (* P-value < 0.05; ** P-value <0.01; *** P-values <0.001). “/” means fermentations were stopped before this time point. (PDF) [file pone.0209124.s006.pdf]

S2 Table. Statistical analysis of the weight loss measured during fermentation between gene edited variants and their respective WT (from time point 1 to end of the fermentation).

	ANOVA					
P-values	H1 vs gene edited H1 variants	H2 vs gene edited H2 variants	BE014 vs gene edited BE014 variants	BE020 vs gene edited BE020 variants	WL022 vs gene edited WL022 variants	WL024 vs gene edited WL024 variants
T1	0.933	0.494	0.032	0.332	0.846	0.295
T2	0.900	0.953	0.071	0.331	0.069	0.068
T3	0.629	0.964	0.104	0.779	0.000***	0.129
T4	0.690	0.760	0.488	0.775	0.008**	0.216
T5	0.418	0.569	0.801	0.975	0.349	0.212
T6	0.973	0.309	0.378	0.593	0.839	0.179
T7	0.747	0.357	0.804	0.711	0.902	0.304
T8	0.783	0.349	0.790	0.677	1.000	0.268
T9	/	0.445	/	/	0.819	0.059
T10	/	0.246	/	/	/	/

P-values were obtained using ANOVA. All statistical analysis were conducted in R, with the multcomp package (* P-value < 0.05; ** P-value <0.01; *** P-values <0.001). "/" means fermentations were stopped before this time point.
