# Supplementary material for: Reducing phenolic off-flavors through CRISPR-based gene editing of the FDC1 gene in Saccharomyces cerevisiae x Saccharomyces eubayanus hybrid lager beer yeasts
Source: PLoS One. 2019 Jan 9;14(1):e0209124. doi: 10.1371/journal.pone.0209124 (PMC6326464; doi:10.1371/journal.pone.0209124)
Supplement: S3 Table — Column two represents the P-values obtained with ANOVA. Column three to twelve represent the obtained P-values of a post-hoc Tukey test. All statistical analyses were conducted in R, within the multcomp package (* P-value < 0.05; ** P-value <0.01; *** P-values <0.001). (PDF) [file pone.0209124.s007.pdf]

S3 Table. Statistical analysis of the phenotypic behavior of H1 compared to the H1 gene-edited variants.

	ANOVA	POSTHOC TUKEY									
P-values	H1 vs gene edited H1 variants	H1_A - H1	H1_B - H1	H1_C - H1	H1_D - H1	H1_B - H1_A	H1_C - H1_A	H1_D - H1_A	H1_C - H1_B	H1_D - H1_B	H1_D - H1_C
**Ethanol**	0.814	0.935	0.449	0.984	0.972	0.210	0.730	1.000	0.689	0.250	0.810
**Glycerol**	0.903	1.000	0.916	0.856	0.997	0.936	0.827	0.994	0.466	0.788	0.957
**SO_2**	0.927	0.645	0.965	0.968	0.822	0.922	0.371	0.235	0.739	0.517	0.989
**Acetaldehyde**	0.859	1.000	0.967	0.970	0.971	0.973	0.964	0.965	0.750	0.752	1.000
**Ethyl acetate**	0.059	0.923	0.444	0.321	0.476	0.824	0.664	0.856	0.997	1.000	0.993
**Ethyl propionate**	0.236	1.000	0.911	0.538	0.430	0.916	0.546	0.437	0.919	0.827	0.999
**Propyl acetate**	0.528	1.000	0.995	0.827	0.962	0.982	0.753	0.921	0.952	0.998	0.993
**Isoamyl alcohol**	0.0389 *	0.467	0.651	0.187	0.618	0.994	0.886	0.997	0.712	1.000	0.745
**isobutyl.acetate**	0.113	0.078	0.490	0.894	0.768	0.457	0.191	0.259	0.900	0.975	0.998
**ethyl.butyrate**	0.612	0.976	0.999	0.943	1.000	0.996	1.000	0.963	0.983	0.998	0.922
**Isopentyl acetate**	0.747	0.396	0.986	0.996	0.944	0.612	0.277	0.190	0.910	0.757	0.995
**Ethyl hexanoate**	0.437	0.202	0.901	1.000	1.000	0.479	0.239	0.167	0.949	0.830	0.996
**Phenethyl alcohol**	0.447	1.000	0.779	0.792	1.000	0.819	0.831	1.000	1.000	0.804	0.817
**Ethyl octanoate**	0.294	0.048	0.518	0.961	1.000	0.255	0.088	0.042	0.832	0.463	0.931
**Phenethyl acetate**	0.341	0.065	0.607	0.976	0.997	0.294	0.112	0.048	0.874	0.456	0.892
**Ethyl decanoate**	0.345	0.719	0.472	0.998	1.000	0.983	0.859	0.731	0.616	0.483	0.998
**4VG**	0.00***	0.005**	0.008**	0.005**	0.005**	0.965	1.000	1.000	0.970	0.982	1.000

Column two represents the P-values obtained with ANOVA. Column three to twelve represent the obtained P-values of a post-hoc Tukey test. All statistical analyses were conducted in R, within the multcomp package (* P-value < 0.05; ** P-value <0.01; *** P-values <0.001).
