# Supplementary material for: Reducing phenolic off-flavors through CRISPR-based gene editing of the FDC1 gene in Saccharomyces cerevisiae x Saccharomyces eubayanus hybrid lager beer yeasts
Source: PLoS One. 2019 Jan 9;14(1):e0209124. doi: 10.1371/journal.pone.0209124 (PMC6326464; doi:10.1371/journal.pone.0209124)
Supplement: S4 Table — Column two represents the P-values obtained with ANOVA. Column three to twelve represent the obtained P-values of a post-hoc Tukey test. All statistical analyses were conducted in R, within the multcomp package (* P-value < 0.05; ** P-value <0.01; *** P-values <0.001). (PDF) [file pone.0209124.s008.pdf]

S4 Table. Statistical analysis of the phenotypic behavior of H2 compared to the H2 gene-edited variants.

	ANOVA	POSTHOC TUKEY									
P-values	H2 vs gene edited H1 variants	H2_A - H2	H2_B - H2	H2_C - H2	H2_D - H2	H2_B - H2_A	H2_C - H2_A	H2_D - H2_A	H2_C - H2_B	H2_D - H2_B	H2_D - H2_C
Ethanol	0.258	0.729	1.000	0.899	0.228	0.729	0.994	0.729	0.899	0.228	0.538
Glycerol	0.774	0.998	0.988	1.000	0.902	0.935	1.000	0.781	0.963	0.993	0.835
SO_2	0.572	0.999	0.426	0.984	0.971	0.516	0.948	0.994	0.258	0.707	0.808
Acetaldehyde	0.187	0.974	0.709	0.493	0.963	0.944	0.774	1.000	0.990	0.958	0.803
Ethyl acetate	0.600	1.000	0.815	0.974	0.999	0.883	0.992	0.995	0.984	0.713	0.926
Ethyl propionate	0.233	0.932	0.395	0.835	0.997	0.754	0.999	0.989	0.872	0.531	0.945
Propyl acetate	0.416	0.931	0.827	0.999	0.999	0.998	0.984	0.984	0.926	0.926	1.000
Isoamyl alcohol	0.412	0.896	1.000	0.960	0.832	0.860	0.999	1.000	0.938	0.791	0.994
isobutyl.acetate	0.725	0.999	0.990	1.000	0.999	0.999	1.000	1.000	0.993	1.000	0.999
ethyl.butyrate	0.176	0.927	0.561	0.854	0.945	0.918	0.999	1.000	0.968	0.895	0.998
Isopentyl acetate	0.558	0.999	0.887	0.990	1.000	0.959	1.000	1.000	0.988	0.908	0.994
Ethyl hexanoate	0.833	1.000	0.958	0.999	0.999	0.945	0.999	1.000	0.989	0.897	0.991
Phenethyl alcohol	0.229	0.733	1.000	0.828	0.522	0.774	0.999	0.991	0.864	0.562	0.964
Ethyl octanoate	0.361	0.998	0.998	0.825	0.834	1.000	0.935	0.941	0.934	0.940	1.000
Phenethyl acetate	0.258	0.951	0.892	0.894	0.963	1.000	1.000	1.000	1.000	0.999	0.999
Ethyl decanoate	0.351	0.935	0.997	0.494	0.726	0.822	0.855	0.983	0.368	0.572	0.987
4VG	0.000***	0.002**	0.001**	0.003**	0.003**	0.871	0.996	0.948	0.709	0.536	0.996

Column two represents the P-values obtained with ANOVA. Column three to twelve represent the obtained P-values of a post-hoc Tukey test. All statistical analyses were conducted in R, within the multcomp package (* P-value < 0.05; ** P-value <0.01; *** P-values <0.001).
