# Supplementary material for: Reducing phenolic off-flavors through CRISPR-based gene editing of the FDC1 gene in Saccharomyces cerevisiae x Saccharomyces eubayanus hybrid lager beer yeasts
Source: PLoS One. 2019 Jan 9;14(1):e0209124. doi: 10.1371/journal.pone.0209124 (PMC6326464; doi:10.1371/journal.pone.0209124)
Supplement: S5 Table — Column two represents the P-values obtained with ANOVA. Column three to twelve represent the obtained P-values of a post-hoc Tukey test. All statistical analyses were conducted in R, within the multcomp package (* P-value < 0.05; ** P-value <0.01; *** P-values <0.001). (PDF) [file pone.0209124.s009.pdf]

**S5 Table. Statistical analysis of the phenotypic behavior of BE014 compared to its gene-edited variants.**

| | ANOVA | POSTHOC TUKEY | | | | | |
|---|---|---|---|---|---|---|---|
| **P-values** | **BE014 vs gene edited BE014** | **BE014 vs BE014_A** | **BE014 vs BE014_B** | **BE014 vs BE014_C** | **BE014_A vs BE014_B** | **BE014_A vs BE014_C** | **BE014_B vs BE014_C** |
| **Ethanol** | 0.138 | 0.741 | 0.790 | 0.284 | 1.000 | 0.712 | 0.661 |
| **Glycerol** | 0.199 | 0.399 | 0.217 | 0.997 | 0.915 | 0.479 | 0.262 |
| **$SO_2$** | 0.675 | 0.989 | 0.885 | 0.368 | 0.748 | 0.487 | 0.184 |
| **Acetaldehyde** | 0.054 | 0.316 | 0.340 | 0.637 | 1.000 | 0.862 | 0.893 |
| **Ethyl acetate** | 0.673 | 0.857 | 0.861 | 0.961 | 1.000 | 0.628 | 0.632 |
| **Ethyl propionate** | 0.206 | 0.910 | 0.063 | 0.695 | 0.111 | 0.961 | 0.172 |
| **Propyl acetate** | 0.630 | 0.741 | 0.979 | 0.996 | 0.909 | 0.632 | 0.928 |
| **Isoamyl alcohol** | 0.250 | 0.858 | 0.436 | 0.950 | 0.810 | 0.992 | 0.678 |
| **isobutyl.acetate** | 0.449 | 0.955 | 0.534 | 1.000 | 0.781 | 0.965 | 0.554 |
| **ethyl.butyrate** | 0.879 | 0.571 | 0.602 | 0.961 | 0.166 | 0.805 | 0.394 |
| **Isopentyl acetate** | 0.690 | 0.924 | 0.994 | 1.000 | 0.982 | 0.928 | 0.995 |
| **Ethyl hexanoate** | 0.961 | 0.973 | 1.000 | 0.983 | 0.981 | 0.866 | 0.976 |
| **Phenethyl alcohol** | 0.753 | 0.997 | 1.000 | 0.962 | 0.998 | 0.990 | 0.967 |
| **Ethyl octanoate** | 0.810 | 1.000 | 0.947 | 1.000 | 0.928 | 0.999 | 0.962 |
| **Phenethyl acetate** | 0.654 | 0.999 | 0.736 | 1.000 | 0.795 | 0.997 | 0.703 |
| **Ethyl decanoate** | 0.891 | 0.989 | 0.992 | 0.992 | 0.934 | 1.000 | 0.943 |
| **4VG** | 0.000*** | 0.029* | 0.029* | 0.027* | 1.000 | 1.000 | 1.000 |

Column two represents the P-values obtained with ANOVA. Column three to twelve represent the obtained P-values of a post-hoc Tukey test. All statistical analyses were conducted in R, within the multcomp package (* P-value < 0.05; ** P-value <0.01; *** P-values <0.001).
