# Supplementary material for: Reducing phenolic off-flavors through CRISPR-based gene editing of the FDC1 gene in Saccharomyces cerevisiae x Saccharomyces eubayanus hybrid lager beer yeasts
Source: PLoS One. 2019 Jan 9;14(1):e0209124. doi: 10.1371/journal.pone.0209124 (PMC6326464; doi:10.1371/journal.pone.0209124)
Supplement: S6 Table — Column two represents the P-values obtained with ANOVA. Column three to twelve represent the obtained P-values of a post-hoc Tukey test. All statistical analyses were conducted in R, within the multcomp package (* P-value < 0.05; ** P-value <0.01; *** P-values <0.001). (PDF) [file pone.0209124.s010.pdf]

S6 Table. Statistical analysis of the phenotypic behavior of BE020 compared to its gene-edited variants.

	ANOVA	POSTHOC TUKEY test					
P-values	BE020 vs gene edited BE020	BE020 vs BE020_A	BE020 vs BE020_B	BE020 vs BE020_C	BE020_A vs BE020_B	BE020A vs BE020_C	BE020_B vs BE020_C
Ethanol	0.560	1.000	0.158	0.987	0.159	0.987	0.117
Glycerol	0.085	0.413	0.647	0.605	0.951	0.970	1.000
SO_2	0.266	0.750	0.088	0.992	0.224	0.875	0.112
Acetaldehyde	0.221	0.674	0.751	0.912	0.998	0.949	0.980
Ethyl acetate	0.774	0.996	0.151	0.489	0.183	0.405	0.040
Ethyl propionate	0.557	0.888	0.010	0.945	0.007	0.998	0.007
Propyl acetate	0.896	0.983	0.477	0.857	0.342	0.969	0.225
Isoamyl alcohol	0.613	0.881	0.835	0.504	0.482	0.253	0.899
isobutyl.acetate	0.706	0.653	0.636	0.534	0.208	0.994	0.165
ethyl.butyrate	0.768	1.000	0.600	0.978	0.552	0.991	0.428
Isopentyl acetate	0.933	0.994	0.943	0.999	0.858	0.999	0.906
Ethyl hexanoate	0.400	0.754	0.418	0.996	0.886	0.650	0.344
Phenethyl alcohol	0.293	0.007	0.546	0.996	0.017	0.008	0.653
Ethyl octanoate	0.836	1.000	0.767	0.971	0.756	0.975	0.555
Phenethyl acetate	0.570	0.682	1.000	0.995	0.690	0.797	0.996
Ethyl decanoate	0.702	1.000	0.987	0.930	0.979	0.909	0.992
4VG	0.000***	0.000***	0.000***	0.000***	0.628	0.625	1.000

Column two represents the P-values obtained with ANOVA. Column three to twelve represent the obtained P-values of a post-hoc Tukey test. All statistical analyses were conducted in R, within the multcomp package (* P-value < 0.05; ** P-value <0.01; *** P-values <0.001).
