# Supplementary material for: Reducing phenolic off-flavors through CRISPR-based gene editing of the FDC1 gene in Saccharomyces cerevisiae x Saccharomyces eubayanus hybrid lager beer yeasts
Source: PLoS One. 2019 Jan 9;14(1):e0209124. doi: 10.1371/journal.pone.0209124 (PMC6326464; doi:10.1371/journal.pone.0209124)
Supplement: S7 Table — Column two represents the P-values obtained with ANOVA. Column three to twelve represent the obtained P-values of a post-hoc Tukey test. All statistical analyses were conducted in R, within the multcomp package (* P-value < 0.05; ** P-value <0.01; *** P-values <0.001). (PDF) [file pone.0209124.s011.pdf]

S7 Table. Statistical analysis of the phenotypic behavior of WL022 compared to its gene-edited variant.

	ANOVA	POSTHOC-TUKEY
P-values	WL022 vs gene edited WL022	WL022 vs WL022_A
Ethanol	0.499	0.499
Glycerol	0.159	0.159
SO_2	0.246	0.246
Acetaldehyde	0.622	0.622
Ethyl acetate	0.154	0.154
Ethyl propionate	0.325	0.325
Propyl acetate	0.225	0.225
Isoamyl alcohol	0.389	0.389
isobutyl.acetate	0.248	0.248
ethyl.butyrate	0.192	0.192
Isopentyl acetate	0.159	0.159
Ethyl hexanoate	0.115	0.115
Phenethyl alcohol	0.390	0.390
Ethyl octanoate	0.866	0.866
Phenethyl acetate	0.087	0.087
Ethyl decanoate	0.636	0.636
4VG	0.025*	0.025*

Column two represents the P-values obtained with ANOVA. Column three to twelve represent the obtained P-values of a post-hoc Tukey test. All statistical analyses were conducted in R, within the multcomp package (* P-value < 0.05; ** P-value <0.01; *** P-values <0.001).
