# Supplementary material for: Reducing phenolic off-flavors through CRISPR-based gene editing of the FDC1 gene in Saccharomyces cerevisiae x Saccharomyces eubayanus hybrid lager beer yeasts
Source: PLoS One. 2019 Jan 9;14(1):e0209124. doi: 10.1371/journal.pone.0209124 (PMC6326464; doi:10.1371/journal.pone.0209124)
Supplement: S8 Table — Column two represents the P-values obtained with ANOVA. Column three to twelve represent the obtained P-values of a post-hoc Tukey test. All statistical analyses were conducted in R, within the multcomp package (* P-value < 0.05; ** P-value <0.01; *** P-values <0.001). (PDF) [file pone.0209124.s012.pdf]

S8 Table. Statistical analysis of the phenotypic behavior of WL024 compared to its gene-edited variant.

	ANOVA	POSTHOC-TUKEY
P-values	WL024 vs gene edited WL024	WL024 vs WL024_A
Ethanol	0.228	0.228
Glycerol	0.054	0.054
SO_2	0.320	0.320
Acetaldehyde	0.969	0.969
Ethyl acetate	0.706	0.706
Ethyl propionate	0.495	0.495
Propyl acetate	0.229	0.229
Isoamyl alcohol	0.004**	0.004**
isobutyl.acetate	0.351	0.351
ethyl.butyrate	0.609	0.609
Isopentyl acetate	0.524	0.524
Ethyl hexanoate	0.266	0.266
Phenethyl alcohol	0.622	0.622
Ethyl octanoate	0.272	0.272
Phenethyl acetate	0.951	0.951
Ethyl decanoate	0.996	0.996
4VG	0.033*	0.033*

Column two represents the P-values obtained with ANOVA. Column three to twelve represent the obtained P-values of a post-hoc Tukey test. All statistical analyses were conducted in R, within the multcomp package (* P-value < 0.05; ** P-value <0.01; *** P-values <0.001).
