# Supplementary material for: Reducing phenolic off-flavors through CRISPR-based gene editing of the FDC1 gene in Saccharomyces cerevisiae x Saccharomyces eubayanus hybrid lager beer yeasts
Source: PLoS One. 2019 Jan 9;14(1):e0209124. doi: 10.1371/journal.pone.0209124 (PMC6326464; doi:10.1371/journal.pone.0209124)
Supplement: S9 Table — Quantified yeast-related aroma compounds are represented as concentrations (mg.L-1), total weight loss as grams (g), ethanol production as volume percentage. Glycerol and SO2 production are represented as concentrations (g.L-1 and mg.L-1 respectively). H2S production capacity is qualitatively indicated (+,+-, -). Lastly, the used score legend for flavors during sensory analysis was: VS = very slightly; S = slightly; V = very; N = neutral; FR = fruity; POF = cloves, phenolic; FRESH = fresh. (PDF) [file pone.0209124.s013.pdf]

S9 Table. Overview aroma and ethanol production from lab scale lager beer fermentation tests.

Strain	total weight loss	ethanol	glycerol	SO2	acetaldehyde	ethyl acetate	ethyl propionate	propyl acetate	isoamyl alcohol	isobutyl acetate	ethyl butyrate	isopentyl acetate	ethyl hexanoate	phenethyl alcohol	ethyl octanoate	phenethyl acetate	ethyl decanoate	4VG	H_2S	Sensorial analysis
W34/70	8.00	6.97	3.13	1.54	9.07	32.62	0.83	0.05	206.23	0.16	0.18	1.93	0.33	95.01	0.86	0.74	0.04	0.26	+-	SFR/FRESH
BE014	7.14	6.11	3.29	1.73	0.25	13.36	0.35	0.01	69.70	0.03	0.07	0.73	0.10	12.11	0.23	0.03	0.53	2.03	-	VFR/SPOF
BE014_A	6.95	6.37	2.83	1.64	1.02	11.83	0.38	0.01	57.14	0.02	0.06	0.60	0.10	11.42	0.23	0.03	0.48	0.00	-	VFR
BE014_B	6.92	6.34	2.67	1.93	0.99	11.84	0.52	0.01	47.97	0.02	0.07	0.68	0.10	11.88	0.26	0.04	0.58	0.00	-	FR
BE014_C	7.43	6.64	3.24	1.20	0.72	14.28	0.40	0.01	59.87	0.03	0.06	0.73	0.10	10.54	0.23	0.03	0.48	0.00	-	FR
BE020	6.45	5.83	2.73	5.93	0.94	11.80	0.25	0.01	77.27	0.02	0.06	0.91	0.06	11.98	0.21	0.06	0.40	1.64	-	VSFR/POF
BE020_A	6.40	5.83	2.46	8.56	0.53	11.68	0.24	0.01	85.43	0.02	0.06	0.95	0.07	19.46	0.21	0.08	0.41	0.06	-	N
BE020_B	6.63	5.92	2.54	14.69	0.58	10.17	0.35	0.01	73.77	0.02	0.05	0.80	0.07	13.36	0.25	0.06	0.35	0.00	-	N
BE020_C	6.48	5.82	2.53	6.64	0.71	12.70	0.24	0.02	69.71	0.02	0.06	0.93	0.06	12.18	0.17	0.06	0.30	0.00	-	N
WL022	6.53	5.76	3.09	7.86	6.43	18.25	0.57	0.02	237.82	0.12	0.08	1.61	0.08	131.01	0.52	1.38	0.14	3.77	+	N/POF
WL022_A	6.56	5.79	3.22	9.53	8.82	21.72	0.45	0.03	238.53	0.14	0.09	1.93	0.11	115.40	0.58	1.59	0.26	0.12	+	SFR
WL024	6.64	5.71	3.20	19.61	13.66	21.05	1.39	0.01	227.34	0.19	0.09	1.68	0.11	129.05	0.87	1.48	0.16	3.70	+	SFR/POF
WL024_A	6.50	5.77	3.55	27.15	13.65	20.66	0.80	0.03	253.85	0.12	0.09	1.58	0.08	136.04	0.39	1.47	0.16	0.00	+	FR
H1	6.62	5.80	2.52	0.94	5.16	24.69	0.68	0.08	218.69	0.13	0.09	2.21	0.11	101.24	0.48	1.13	0.03	3.25	+-	SFR/SPOF
H1_A	6.49	5.85	2.56	1.06	5.14	23.68	0.69	0.08	199.72	0.17	0.09	2.82	0.14	107.37	1.06	1.61	0.18	0.00	+-	VFR
H1_B	6.59	5.69	2.94	0.99	3.45	22.34	0.82	0.07	206.35	0.15	0.09	2.39	0.12	155.46	0.74	1.35	0.23	0.10	+-	VFR
H1_C	6.63	5.77	2.03	0.89	7.84	23.29	1.00	0.07	202.99	0.15	0.09	2.27	0.12	165.15	0.63	1.30	0.08	0.00	+-	VFR
H1_D	6.64	5.84	2.37	0.86	6.52	21.24	0.94	0.07	195.16	0.14	0.09	1.91	0.10	101.99	0.45	1.05	0.04	0.00	+-	VFR
H2	7.13	6.23	2.69	1.23	4.62	24.64	0.92	0.04	223.01	0.12	0.11	1.71	0.16	104.91	0.67	0.75	0.21	3.71	+	FR/POF
H2_A	7.16	6.29	2.67	1.27	3.71	25.25	0.78	0.05	215.30	0.12	0.12	1.84	0.16	162.17	0.61	0.95	0.15	0.05	+	FR
H2_B	6.82	6.23	2.73	1.64	1.93	25.64	0.50	0.05	206.99	0.12	0.12	1.98	0.18	103.14	0.56	0.91	0.22	0.00	+	SFR
H2_C	6.99	6.27	2.68	1.12	1.44	25.38	0.70	0.04	209.39	0.11	0.12	1.85	0.16	147.83	0.46	0.94	0.07	0.15	+	FR
H2_D	7.18	6.36	2.76	1.36	3.84	25.28	0.90	0.05	223.89	0.13	0.13	1.86	0.16	199.46	0.51	1.00	0.12	0.43	++	FR

Quantified yeast-related aroma compounds are represented as concentrations ($mg.L^{-1}$), total weight loss as grams (g), ethanol production as volume percentage. Glycerol and SO_2 production are represented as concentrations ($g.L^{-1}$ and $mg.L^{-1}$ respectively). H_2S production capacity is qualitatively indicated (+,+-, -). Lastly, the used score legend for flavors during sensory analysis was: VS = very slightly; S = slightly; V = very; N =neutral; FR = fruity; POF = cloves, phenolic; FRESH = fresh.
