# Supplementary material for: Reducing phenolic off-flavors through CRISPR-based gene editing of the FDC1 gene in Saccharomyces cerevisiae x Saccharomyces eubayanus hybrid lager beer yeasts
Source: PLoS One. 2019 Jan 9;14(1):e0209124. doi: 10.1371/journal.pone.0209124 (PMC6326464; doi:10.1371/journal.pone.0209124)
Supplement: S10 Table — (PDF) [file pone.0209124.s014.pdf]

S10 Table. Overview used primers

Primer	Sequence (5' to 3')	use
SS_FWSC	AGAATTGCCCATCATCTGGG	*S. cerevisiae* specific forward sanger sequencing primer
SS_RVSC	ACCTTCAGGAATTGGCATGG	*S. cerevisiae* specific reverse sanger sequencing primer
SS_FWSE	GAATTGCTCATCATCTCGGG	*S. eubayanus* specific forward sanger sequencing primer
SS_RVSE	TCGCCCAAAATTGCACCGAT	*S. euba0yanus* specific reverse sanger sequencing primer
RT_FWSC	ATACATCTACAAAGCCTGCCAACACCATATCTACATGTTTCAGACGGTGGCAAGTACTTA	*S. cerevisiae* specific forward primer for the generation of the repair template
RT_RVSC	TTTTATCTGGAGTTTGAAGAATCCACATTCCGTACGTTTATAAGTACTTGCCACCGTCTG	*S. cerevisiae* specific reverse primer for the generation of the repair template
RT_FWSE	GTTCATTTAGATAGTCTTCCAGCACCATACTTGCATACTTCTGATGGTGGCAAGTATTTG	*S. eubayanus* specific forward primer for the generation of the repair template
RT_RVSE	TCTTATCTGGAGTTTGAAGAATCCACATTCCGTACGTTTACAAATACTTGCCACCATCAG	*S. eubayanus* specific reverse primer for the generation of the repair template
FW1	CCCTCTTTCTCTTTGCTTTC	See FIG S2
RV1	AGTAGAGAGGGCATAGATCG	See FIG S2
FW2	GAATTGCTCATCATCTCGGG	See FIG S2
RV2	TTGCCACCATCAGAAGTATG	See FIG S2
FW3	GGAAGACGTATGGCTACAAG	See FIG S2
RV3	CTGTTCTTTCCTGTTATCGC	See FIG S2
